# Recent Trends in Artificial SEI Layers for Controlling Dendrite Formation and Enhancing Cycle Life: Toward Stable and Durable Sodium Metal Batteries

**DOI:** 10.1002/smll.202502974

**Published:** 2025-07-17

**Authors:** Megala Moorthy, Ranjith Thangavel, So‐Yeon An, Anjali Anilkumar, Daehoon Han, Yun Sung Lee

**Affiliations:** ^1^ School of Chemical Engineering Chonnam National University Gwangju 61186 Republic of Korea; ^2^ Department of Chemical Engineering Indian Institute of Technology Tirupati Tirupati 517619 India

**Keywords:** artificial SEI layer, dendrites, homogenous deposition, interphase strength, long lifespan, sodium metal batteries

## Abstract

The growing demand for sustainable energy storage solutions has stimulated extensive research efforts on sodium metal batteries (SMBs), as a promising alternative storage device to lithium‐based systems. SMBs provide advantages over high theoretical capacity and cost‐effectiveness, as well as widely available sodium resources, which effectively address the critical limitations associated with the scarcity and high cost of lithium. However, the development of SMBs is hindered by several challenges such as nonuniform sodium ion deposition, dendrite formation, and unstable solid electrolyte interphase (SEI) layers, which lead to poor cycle life and safety concerns. This review provides a comprehensive analysis of the fundamental challenges associated with sodium metal anodes, focusing on the mechanisms of sodium dendrite growth and SEI layer formation. The need for several types of artificial SEI layers and corresponding formation strategies, highlighting their advantages in preventing dendrite formation, is also explored. Furthermore, this review emphasizes the role of advanced characterization techniques, particularly using in situ and cryogenic tools, in elucidating the working mechanisms of sodium metal anodes. Overall, this work aims to provide in‐depth insights into the critical bottlenecks and prospective solutions, offering a forward‐looking perspective on the advancement of sodium metal battery technologies.

## Graphical abstract

This review discusses the significance of Na Metal Anodes for Sodium Metal Batteries, highlighting key challenges such as dendrite growth, unstable SEI, and gas evolution. It also explores various protective layers for dendrite‐free Na, including organic SEI layers, inorganic SEI layers, and hybrid SEI layers, emphasizing the role of interphase engineering. Advanced characterization techniques and structural modifications are analyzed to enhance battery performance and safety.

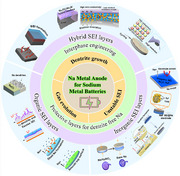

## 1 Introduction

### 1.1 Relentless Research on Sodium Metal Anodes for SMBs

**Figure 1:**
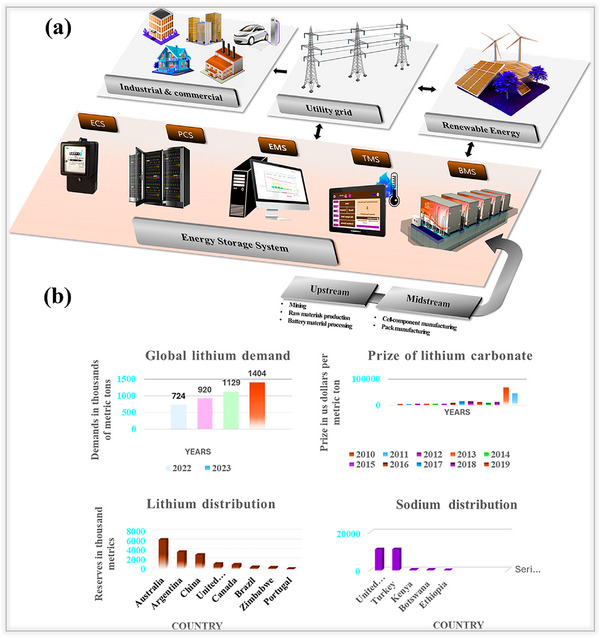
a) Overview of energy storage systems supporting industrial, commercial, and grid storage along with worldwide advances. b) Global lithium demand. Reproduced with permission^[^ ^8^ ^]^ Copyright,2024 Statista. Rising price trends of lithium carbonate. Reproduced with permission^[^ ^10^ ^]^ Copyright,2024 Statista. Worldwide distribution of lithium Reproduced with permission^[^ ^12^ ^]^ Copyright,2024 Statista. and Sodium resources. Reproduced with permission.^[^ ^13^ ^]^ Copyright,2024 Statista.

The storage of energy generated from natural resources such as wind, solar power, and fossil fuels faces significant challenges, owing to the climate change concerns and high production costs. As the global population continues to grow, the escalating demand for energy necessitates the development of efficient storage systems capable of preserving energy in an environmentally sustainable manner, without contributing to global pollution.^[^ ^1^, ^2^, ^3^ ^]^ In this context, electric vehicles are expected to play a pivotal role, in reducing fossil fuel consumption and greenhouse gas emissions. To meet the future energy needs, both China and the United States have set ambitious targets for the energy density of EVs, aiming to reach 500 Wh kg^−1^ by 2030. Additionally, the US Department of Energy has stated that a commercially viable EV must be capable of running 500 km on a single charge.^[^ ^4^ ^]^ To achieve these targets, storage devices should possess high energy and power densities, as well as a long cycle life, for both portable and grid‐scale applications, as illustrated in **Figure** **1**a,b. Lithium‐ion batteries (LIBs) have been widely commercialized since 1990, effectively supporting various applications for over three decades and becoming integral to our daily lives.^[^ ^5^, ^6^, ^7^ ^]^ However, with increasing demand for LIBs, lithium production may fall short of market expectations. Because this metal is relatively scarce, constituting only 0.0017% of the Earth's crust^[^ ^8^, ^9^ ^]^ Moreover, lithium resources are geographically concentrated in a few regions, including Chile, Australia, and China, rather than being evenly distributed worldwide. This limited supply leads to high extraction costs, which are expected to rise further in the coming years.^[^ ^10^ ^]^ With these limitations, large‐scale electricity storage and EV development may struggle with sustainability if they continue to rely on lithium‐based systems. These challenges highlight the urgent need for alternative energy storage systems.^[^ ^11^ ^]^ The key requirements for alternative systems include affordability and wide availability, enabling the development of high‐energy‐density rechargeable batteries. Sodium‐ion batteries (SIBs) are emerging as a more sustainable alternative. Sodium is abundantly available, constituting 2.3% of the Earth's crust, and is significantly more cost‐effective than lithium. Unlike lithium, sodium is widely distributed across multiple countries, including the United States and Turkey, which hold over 11 million tons of sodium carbonate reserves. Additionally, sodium is abundant in seawater, making its production comparatively easier and more accessible than that of lithium.^[^ ^12^, ^13^ ^]^ These attributes position SIBs as strong candidates for future energy storage systems. Although sodium‐ion batteries (SIBs) share a similar electrochemical reaction mechanism with lithium‐ion batteries (LIBs), substantial research is still needed to optimize their performance and commercial viability. Notable progress has been achieved in the development of cathode materials, while several anode materials have also been actively investigated. Conventional SIB anodes, such as hard carbon, alloying materials, and intercalation‐type anodes, provide reasonable electrochemical performance but struggle to achieve high energy/power densities.^[^ ^14^, ^15^, ^16^, ^17^, ^18^, ^19^, ^20^ ^]^

Sodium metal has emerged as a highly promising anode for developing high‐energy batteries. Like lithium, sodium metal belongs to the Group IA alkali metals and exhibits similar physical and chemical properties. It possesses an exceptional theoretical capacity of 1166 mAh g^−1^ and a low chemical reduction potential, low density, which makes it a strong candidate for next‐generation sodium metal and solid‐state batteries.^[^ ^16^, ^21^, ^22^ ^]^ Additionally, Al foil can be used as a current collector instead of copper, because aluminum does not react with sodium and it's a more cost‐effective material. However, despite these advantages, the development of sodium metal batteries (SMBs) faces critical challenges. One of the most significant issues is nonuniform sodium ion(Na^+^) deposition, which leads to dendrite formation and limits battery cycle life.^[^ ^19^, ^23^ ^]^ Addressing these issues is critical for producing sodium metal batteries as viable energy storage systems for EVs and other portable devices.^[^ ^24^ ^]^ Compared to sodium‐ion batteries, sodium metal batteries deliver superior performance, particularly in room‐temperature Na–CO_2_,^[^ ^25^ ^]^ Na–O_2_,^[^ ^26^ ^]^ and Na–S^[^ ^27^ ^]^ systems. Nevertheless, sodium metal is highly reactive and thermodynamically unstable, leading to several issues such as volume expansion and contraction, detachment of alkali ions(Na ^+^) from the bulk phase, gas evolution in the electrolyte, and breakage of the solid electrolyte interphase (SEI).^[^ ^28^, ^29^, ^30^ ^]^ Various solutions have been proposed to address dendrite‐related issues associated with sodium metal anodes, including the design of 2D current collectors^[^ ^31^ ^]^ to regulate uniform plating/stripping and artificial SEI layers for passivating metal from side reactions,^[^ ^32^, ^33^, ^34^ ^]^ 3D carbon‐based^[^ ^35^ ^]^ or metal‐based current collectors^[^ ^36^ ^]^ with sufficiently large surface area to accommodate sodium ions and reduce the local current density.^[^ ^37^ ^]^ However, these host‐type anodes require pre‐sodiation before battery assembly, making them less practical. Then, electrolyte modifications, such as optimizing the salt concentration or using additives to stabilize the SEI. However, liquid electrolyte components are often sacrificed during the initial cycles, making it challenging to achieve long‐term cycle stability.^[^ ^11^ ^]^ Among the proposed solutions, artificial SEI layers are considered the most promising systems because they can be prepared through physical or chemical coating directly on the metal anode, providing a simpler and more effective approach. Artificial SEI layers are classified as organic, inorganic and hybrid, based irrespective  their components. A well‐designed artificial SEI layer must exhibit stability, homogeneity, high ionic conductivity, mechanical strength, uniform thickness, and superior kinetic properties in order to extend the battery's lifespan and enhance its safety.^[^ ^38^, ^39^, ^40^ ^]^

This review paper aims to systematically address the critical aspects of sodium metal anodes in the context of metal battery development. First, we outline the key challenges associated with the use of sodium metal, focusing on its fundamental limitations and the underlying mechanisms of dendrite formation. Second, we summarize and critically evaluate various strategies for the formation of artificial solid electrolyte interphase (SEI) layers, emphasizing their effectiveness in enhancing electrochemical performance. Third, we highlight the use of both conventional and advanced characterization techniques, including in situ and cryogenic tools, to investigate the interfacial behavior and working mechanisms of sodium metal anodes. Finally, we present the key insights gained from this review and provide future perspectives on the practical implementation of sodium metal anodes in next‐generation storage systems.

### 1.2 History of Sodium Metal Batteries

The development of sodium metal–sulfur(Na‐S) battery systems began in the early 1960s, aimed to address the increasing energy demands expected for the future. These batteries operated at high temperatures, ≈300 °C, utilizing molten sodium as the anode, a sulfur cathode, and a beta‐alumina solid‐state electrolyte, known for its high ionic conductivity. Although these high‐temperature batteries demonstrated significant potential, they still faced critical challenges, including frequent maintenance requirements and complex safety measures. These issues resulted not only in complex operations but also in increased costs, hindering their large‐scale commercialization.^[^ ^41^, ^42^ ^]^ Similar to LIBs, early SIBs encountered significant challenges over the last decade, particularly the absence of suitable anode materials. However, substantial progress has been made toward commercializing sodium‐ion technologies, driven by the abundance, low cost, and wide geographical availability of sodium(Na). These characteristics make sodium‐based energy storage systems a promising option for next‐generation energy storage.^[^ ^14^ ^]^ In particular, an increasing amount of research has focused in recent years, coinciding with the rapid growth of studies on sodium‐ion batteries since 2009. Unlike LIBs, which rely on graphite as the primary anode material, sodium‐based batteries cannot use graphite due to its lower capacity. Hence, alternative anode materials such as hard carbon, metal oxides, and sulfides have been explored. Obtaining storage devices with high energy density requires an anode with high theoretical capacity and low overpotential, combined with a cathode providing both high capacity and excellent potential.^[^ ^28^, ^43^ ^]^ Recent advances have led to the development of room‐temperature sodium metal batteries using liquid electrolytes. Although sodium metal anodes hold significant promise, their working chemistry is still unclear. To enable the safe and efficient use of sodium metal batteries, it is crucial to address the associated challenges, particularly from a technoeconomic perspective. Continued research and innovation in this field will be the key to unlocking the potential of sodium metal anodes and establishing them as an essential component of next‐generation energy storage technologies.^[^ ^19^ ^]^

### 1.3 Intrinsic Properties of Li/Na Metal

**Figure 2:**
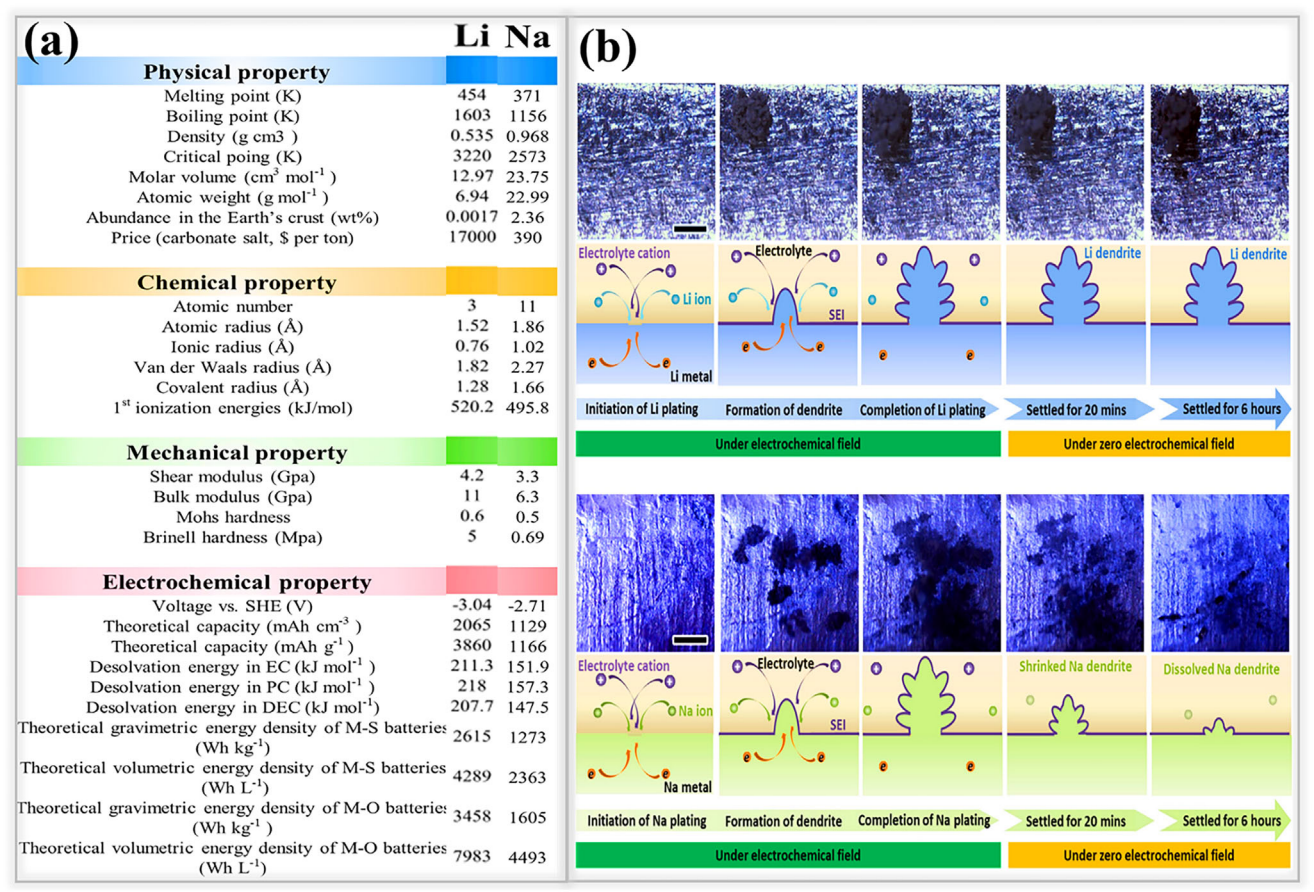
a) Internal properties of lithium and sodium metal anodes. b) Dendrite formation and changes on lithium/sodium metal observed during the plating/stripping process at 0.4 mA cm^−2^. Reproduced with permission^[^ ^47^ ^]^ Copyright 2018, Elsevier, Dendrites were analyzed under both electrochemical and quasi‐zero electrochemical field conditions.

Sodium metal belongs to Group IA in the periodic table, and it is positioned below lithium, sharing several physical and chemical characteristics with it, as shown in **Figure** **2**a. In general, all Group IA elements possess a single electron in their outermost orbital, which can be easily released to form chemical bonds with neighboring atoms. Due to the relatively large atomic radius, the outer electron in sodium is weakly attracted to the nucleus, resulting in low ionization energy. This renders sodium highly reactive, particularly in the presence of polar solvents or electrolytes. Upon contact with an electrolyte, sodium metal readily undergoes oxidation, releasing electrons that subsequently drive reduction reactions at the metal/electrolyte interface.^[^ ^44^, ^45^ ^]^ Owing to its high reactivity, sodium metal forms an unstable natural SEI layer with insufficient mechanical stability. So, it lacks the structural integrity required to suppress dendritic growth and accommodate the significant volume changes that occur during continious plating and stripping cycles.^[^ ^46^ ^]^ Numerous investigations have been conducted on lithium and sodium metal dendrites under active cycling conditions or after cycling. In these studies, the cells were dismantled to examine the morphology of the dendrites. Hong and their team were the first to conduct experiments aimed at observing the dendrite morphology, under a quasi‐electrochemical field (where the cell is halted for some time without any electron transfer movement) and analyze the morphology using *operando* optical microscopy, as shown in Figure 2b. For these experiments, a transparent chamber was constructed to observe the ion deposition on the metal during the cycling process. To compare both lithium and sodium dendrites, they assembled in the chamber with 1 M LiPF_6_ and 1 m NaPF_6_ dissolved in the respective solvents (dimethyl carbonate (DMC)/ethylene carbonate (EC) in a 1:1 ratio). Initially, metal ions were plated on their respective electrodes at a current density of 0.4 mA cm^−2^. As expected, the deposited ions had an uneven and rough appearance due to the high energy barrier in their pristine state, which is a key factor in the nucleation and dendrite formation processes in both cells. After plating, both cells were left undisturbed. Following this interval, the results showed that lithium dendrites remained visible and unchanged, with no significant differences before or after the resting period. In contrast, sodium dendrites began to dissolve over time, with a significant portion disappearing after 6 h. This behavior is attributed to the uneven SEI layer formation in the sodium cells and the poor mechanical stability of the SEI.^[^ ^47^ ^]^

### 1.4 History and Principle of SEI Layer

**Figure 3:**
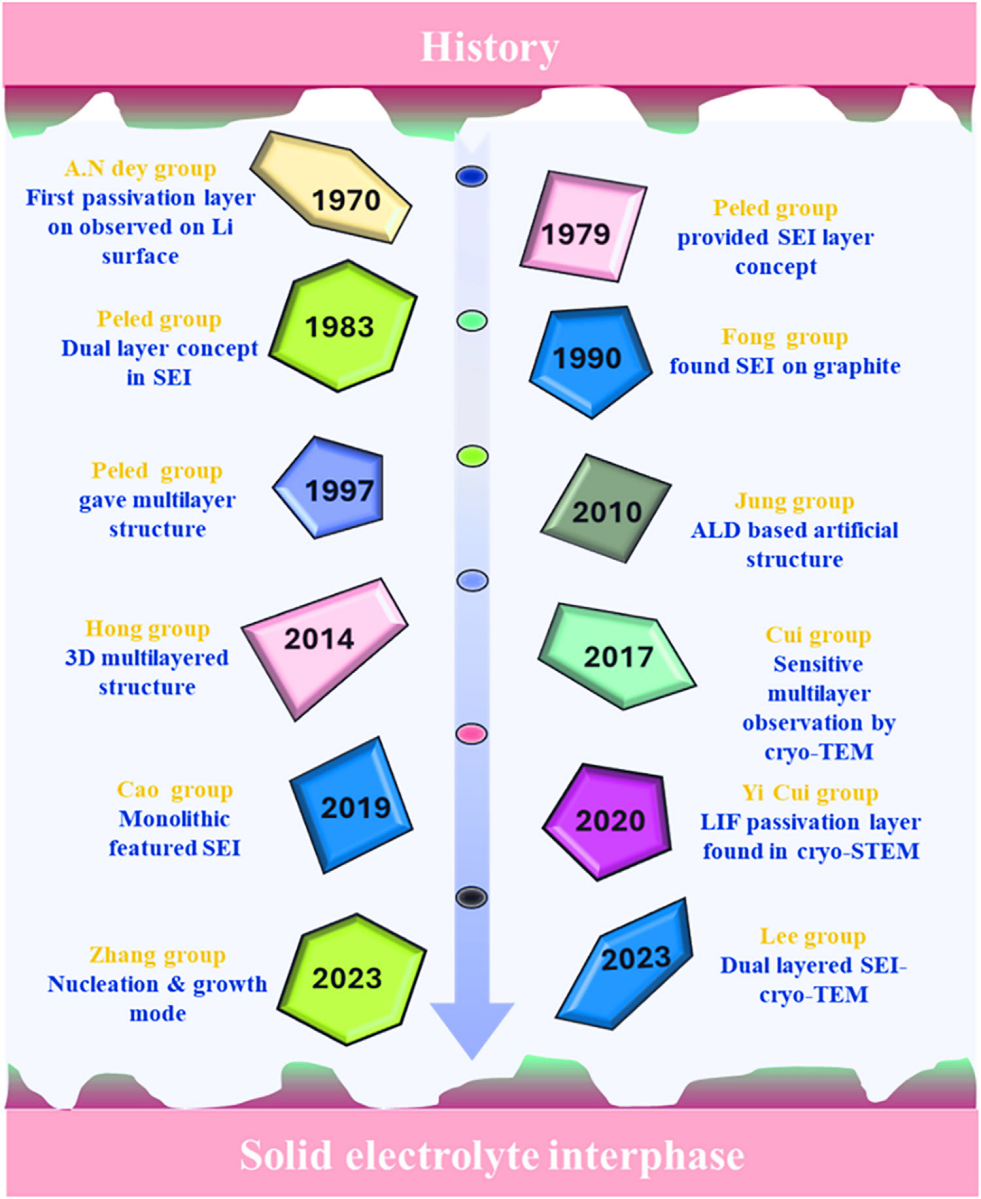
Timeline of solid electrolyte interphase layer discovery and advances from 1970 to 2023.

The SEI layer was first studied by Peled et al. in the 1970s. Later, Peled and Abruch et al. conducted further research to gain a deeper understanding of SEI layers, aiming to develop safer rechargeable lithium‐ion batteries.^[^ ^48^ ^]^ The progress in the SEI layer research over the past 50 years is summarized in **Figure** **3**. Initially, the natural SEI layer forms at the interface between the metal and electrolyte during the first cycle. The main components of the SEI layers are originate from the electrolyte, including conducting salt and organic solvent, while external factors such as current density, areal capacity, temperature, and surface potential of the lithium electrodes also play an important role.^[^ ^44^ ^]^ The ideal characteristics of the SEI layer are as follows: 1)the SEI must be ionically conductive and electronically insulating to ensure smooth ion transport and prevent electron penetration. 2) It should have optimized thickness to reduce the charge transfer resistance, because an excessively thick layer will increase the ion migration barrier. 3) The structure should be highly stable and uniform to maintain an even lithium‐ion deposition over long period of time. 4) Last, but most important criterion is that the formed SEI layer should be mechanically strong to efficiently reduce stresses, such as volume expansion/contraction, damage to the SEI at high rates. Particularly, those impacts are caused by vertical dendrite growth, which can penetrate the opposite electrode and cause short circuits.^[^ ^49^ ^]^

### 1.5 Formation and Composition of SEI Layer

**Figure 4:**
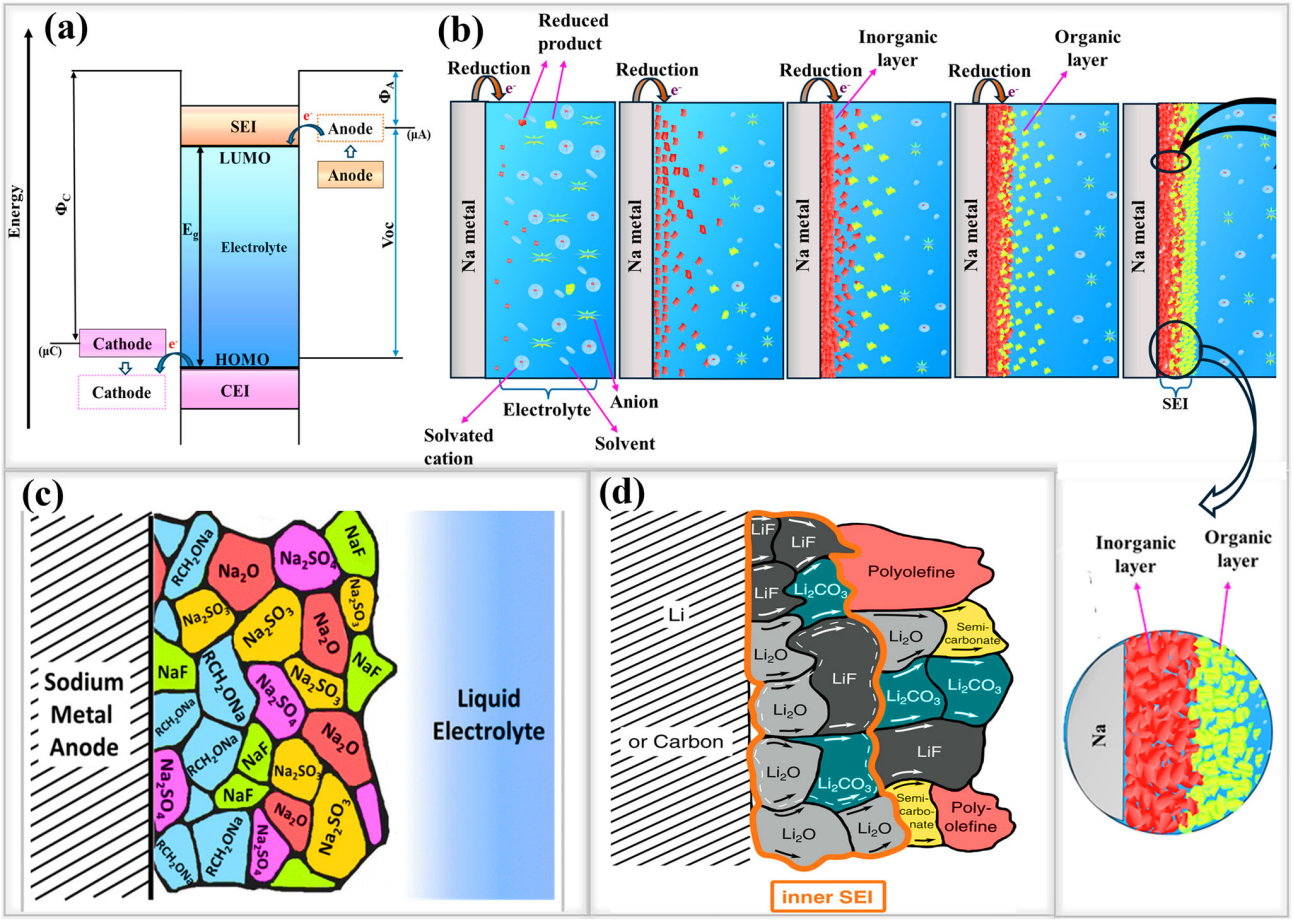
a) Illustration of SEI formation process based on molecular orbital theory. b) Step‐by‐step illustration of electrolyte reduction process to form inorganic and organic SEI layers on sodium metal anode. c) Reduced components on sodium metal anode. Reproduced with permission.^[^ ^43^ ^]^ Copyright 2024, RSC Publishing d) Reduced components on lithium metal anodes. Reproduced with permission^[^ ^55^ ^]^ Copyright 2022, Wiley ‐VCH Publishing.

Goodenough et al, proposed the SEI layer formation mechanism in terms of molecular orbital theory, in which the highest occupied molecular orbital (HOMO) and lowest unoccupied molecular orbital (LUMO) energy levels of the electrolyte plays a key role. The LUMO represents the energy level where empty states can accept electrons, the HOMO is the energy level where electrons are stored. The LUMO and HOMO energy levels of the electrolyte are separated by an energy gap (*E*_g_), as shown in **Figure** **4**a. If the anode potential (µA) is higher than the LUMO energy level of the electrolyte, electrons are transferred from the anode to the electrolyte. These electrons react with solvent species, initiating reduction reactions and leading to the formation of the SEI layer on the anode. Similarly, if the cathode potential (µC) is lower than the HOMO energy level of the electrolyte, electrons are transferred from the electrolyte to the cathode, which triggering oxidation reactions. This results in the formation of the cathode electrolyte interphase(CEI) layer.^[^ ^50^ ^]^ The structure of the natural SEI layer primarily forms through the reduction of the electrolyte components, which contains conducting salts and organic solvents. The step‐by‐step mechanism of SEI layer formation on sodium metal anodes is illustrated in Figure 4b. When the potential of sodium(Na) is higher than the LUMO level of the electrolyte, at this point electrons transfer to the electrolyte and form a double layer in a step‐by‐step manner.^[^ ^51^ ^]^ The resulting SEI layer formed on the sodium metal anode consists of inorganic components (Na_2_O, NaF, Na_2_CO_3_, etc.) near the sodium metal and organic components (sodium alkoxides such as ROCO_2_Na, RCOO_2_Na, and RONa, etc) closer to the electrolyte, as shown in Figure 4c. These products also originate from various sodium‐ion‐conducting salts, such as NaPF_6_(Sodium hexafluorophosphate), NaClO_4_ (Sodium perchlorate), and NaTFSI Sodium bis(trifluoromethanesulfonyl)imide, in combination with organic solvents such as ethylene carbonate (EC), diethyl carbonate (DEC), dimethyl carbonate (DMC), propylene carbonate (PC), tetraethylene glycol dimethyl ether (TEGDME), and tetrahydrofuran (THF).^[^ ^43^ ^]^ Once the double layer is formed, further electron transfer to the electrolyte for additional decomposition becomes difficult.^[^ ^52^ ^]^

Similar conducting salts such as LiPF_6_(Lithium hexafluorophosphate), LiTFSI (Lithium bis(trifluoromethanesulfonyl)imide), and LiClO_4_ (lithium perchlorate) are used in lithium systems, along with organic solvents such as EC, PC, DMC, DEC, ethyl methyl carbonate (EMC), TEGDME, and THF. Similar to sodium systems, the natural SEI formed on lithium consists of both organic and inorganic components arranged in a mosaic structure, as shown in Figure 4d. The layer closer to the lithium metal anode is the inorganic SEI components, primarily composed of LiOH, Li_2_CO_3_, and LiF. The layer near the electrolyte is an organic component, consisting of species originating from organic solvents, such as ROCO_2_Li and RCOO_2_Li, ROLi.^[^ ^53^, ^54^, ^55^ ^]^

### 1.6 Dendrite Formation Mechanisms on Sodium Metal

**Figure 5:**
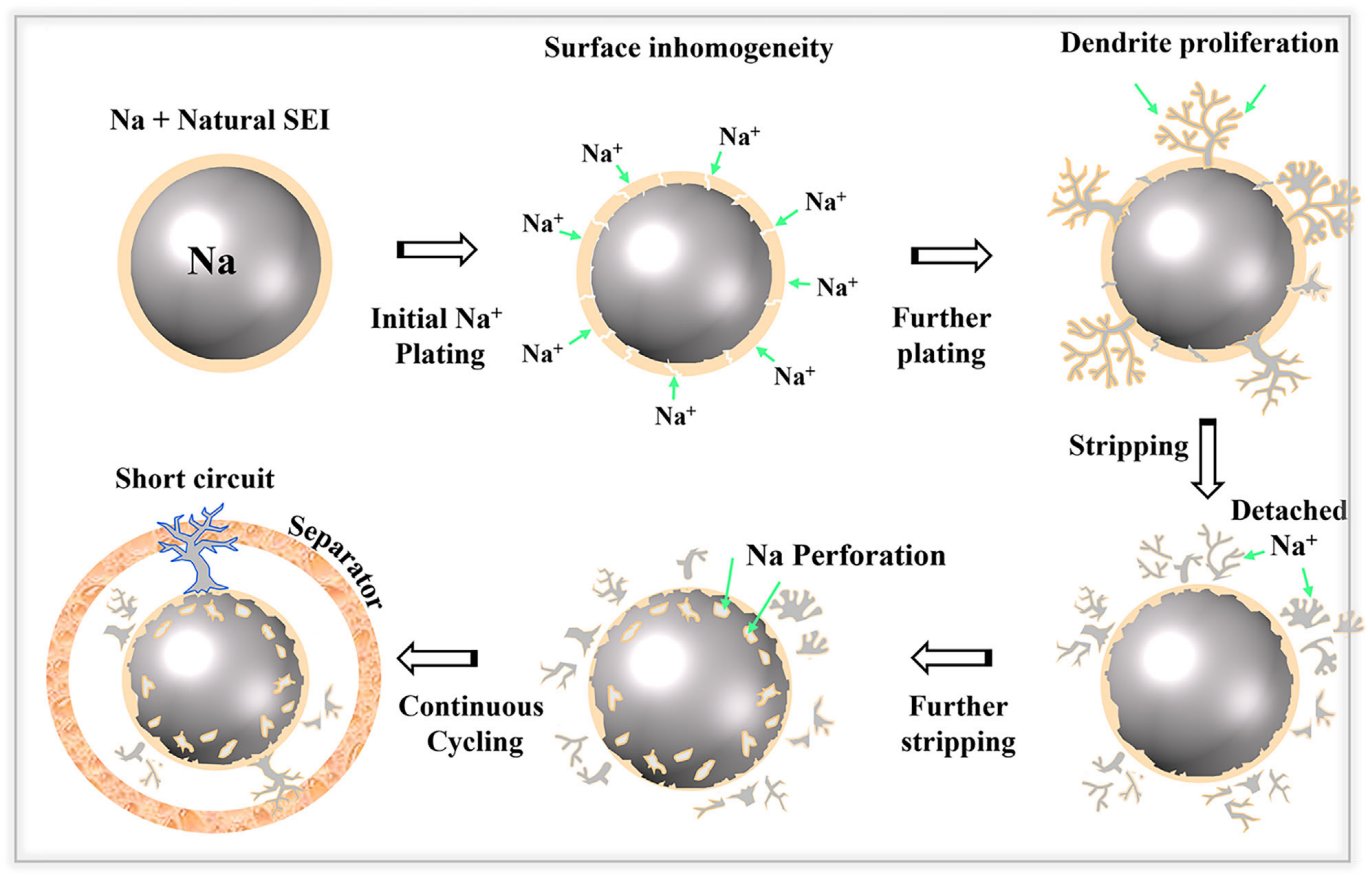
Schematic illustration of dendrite formation process on sodium metal anodes.

Sodium metal anodes provide several advantages that can enhance the lifespan of SMBs. However, dendrite formation remains one of the greatest challenges during the cycling process, significantly limiting their practical application in next‐generation sodium batteries.^[^ ^54^ ^]^ Dendrites generally form in two main structures: vertically and horizontally. These structures can adopt various shapes, including tree‐like, mossy like, snowflake, tube‐like, leaf‐like, and mushroom‐like forms, depending on the respective materials and their operating conditions.^[^ ^56^, ^57^, ^58^ ^]^ **Figure** **5** illustrates the dendrite formation mechanism associated with unstable SEI layers, even at low current densities. During the initial plating process on the sodium metal anode, the unstable SEI layer leads to uneven sodium (Na^+^) deposition. Over time, repeated cycling causes the metal to expand and contract significantly, which can break the unstable SEI layer. When this occurs, fresh sodium metal comes into direct contact with the electrolyte, triggering repeated electron transfer reactions again. The roots of the dendrites may also break off into the electrolyte, making the sodium ions inactive as dead Na^+^ species. Then, the non‐uniform deposition leads to the formation of surface protrusions and deformed anodes. In the final stages, dendrites can grow and penetrate the separator, inducing electrical contacts between the anode and cathode, which can lead to short‐circuiting.^[^ ^19^, ^23^ ^]^ The charge density is inversely proportional to the surface curvature of radius. Structures with smaller curvature radii, such as sharp protrusions, have a higher charge density compared to flatter surfaces with larger radii. These types of sharp needle‐like structures attract more ions and exhibit higher charge concentrations.^[^ ^59^, ^60^, ^61^ ^]^ Dendrite formation on the metal anode begins at Sand's time, defined as:

(1) $$\tau Sand^{\prime }s\;time=\frac{\pi D{\left(Coe\right)}^{2}}{{\left(2Jt_{a}\right)}^{2}}$$

where *D* is the diffusion constant, *Co* is the cation concentration in the electrolyte, *e* is the elementary positive charge, *J* is the applied current density, and *t*_a_ is the transference number of the anions involved in the reactions. The Sand's time (*τ_t_*) parameter represents the critical moment when dendrite growth begins within the system. At this time, cation concentration near the electrode surface decreases to zero, causing a concentration gradient‐driven depletion region. At this stage, the electrolyte can no longer sustain to maintain the uniform ion transport, and dendritic deposition of sodium metal is initiated due to the local supersaturation of cations at the electrode/electrolyte interface. By manipulating factors, such as ion concentration and current density, dendrite growth can be suppressed or delayed. For example, decreasing the current density effectively extends the Sand's time, thereby minimizing dendrite nucleation and enhancing the stability of the system.

## 2 Dendrite‐Related Problems and Challenges

In this section, we discuss the three main dendrite‐related problems and their impact on the capacity of the metal anodes.

### 2.1 Unstable SEI

The deposition (plating) and dissolution (stripping) of sodium ions (Na^+^) on metal anodes lead to various changes. During the plating process, sodium ions are deposited onto the anode and, by receiving electrons via a redox reaction, they form sodium atoms. Conversely, in the stripping process, these deposited atoms return to their original ionic state by releasing the electrons.^[^ ^43^, ^62^ ^]^ However, inefficient stripping (the incomplete removal of Na⁺ ions) arises primarily due to the presence of a heterogeneous and unstable naturally formed SEI layer. As a result, residual sodium tends to accumulate locally at specific regions on the anode surface. With continuous cycling, these localized deposits act as nucleation sites for subsequent sodium deposition, leading to nonuniform stacking of sodium species and ultimately promoting dendrite growth. Moreover, some sodium atoms that fail to be fully stripped during the dissolution process may either nucleate within the SEI matrix or detach into the electrolyte, becoming electrochemically inactive. These inactive sodium species are commonly referred to as dead sodium, and their presence contributes to reduced Coulombic efficiency and long‐term capacity fade.^[^ ^21^, ^23^ ^]^

### 2.2 Volume Expansion/Contraction

Volume expansion issues are more severe in sodium than lithium metal anodes, because sodium has a density of 0.968 g cm^−3^ and a molar mass of 22.99 g mol^−1^, which are higher than those of lithium(0.535 g cm^−3^ and 6.94 g mol^−1^).^[^ ^63^ ^]^ Unlike intercalation‐type anodes, sodium metal anodes have a host‐less nature, which makes them undergo infinite volume expansion/contraction. Upon contact with the electrolytes during the first charge, the thermodynamically unstable nature of sodium metal leads to the formation of an unstable natural SEI layer. The initially formed protective layer is ionically conductive and electronically resistive, but cannot survive for the long‐term plating/stripping process.^[^ ^64^, ^65^ ^]^ With continuous cycling, the natural SEI becomes weak, leading to unstable sodium deposition. Over time, the uneven surface forms protrusions, causing the sodium metal to expand significantly. In extreme cases, this expansion breaks the SEI layer, allowing sodium metal to transfer electrons to the electrolyte. Then, the electrolyte components gain electrons and undergo reduction reactions. Both the unstable SEI and the growth of uncontrolled sodium dendrites play major roles in the expansion of sodium metal anodes.^[^ ^15^ ^]^ This volume expansion results not only in an increased size of the sodium metal anode, but also in the generation of additional side products, which in turn leads to an increased charge transfer resistance. Moreover, sodium metal also has a low yield strain, implying that it deforms under a specific level of stress. Once this stress exceeds the limit, the structure of the sodium metal changes permanently. Therefore, materials with a high yield strain or a 3D host must be integrated into sodium metal anodes to accommodate the large volume expansion without causing structural damage.^[^ ^64^ ^]^

### 2.3 Gas Evolution Inside Battery

Many studies suggest that sodium metal anodes have similar characteristics to lithium metal anodes, but their properties present some key differences. Sodium is highly reactive, making it more prone to react with electrolytes.^[^ ^66^ ^]^ The higher reactivity can result in a less stable natural SEI layer and sometimes lead to gas evolution. To fully understand the problem, it is important to clarify, how gas evolution takes place, what types of gases are produced, and how they impact the anode, cathode, and other components. One common cause is the presence of impurities in the electrolyte, such as moisture. Even trace amounts of water can react with solvent molecules in non‐aqueous electrolytes, producing gaseous byproducts within the cell. Hence, it is crucial that the electrolyte be rigorously dried and free from residual surface moieties. In addition, an unstable SEI layer can fail to passivate the sodium surface effectively, leading to continuous decomposition of electrolyte components and the release of gaseous species. The most reported gases include hydrogen (H_2_), carbon dioxide (CO_2_), and methane (CH_4_). Then, high current densities can make the problem worse by promoting sodium dendrite growth, which triggers additional side reactions and leads to increased gas formation. Once gas starts forming inside the battery, it creates pressure that can damage both the anode and cathode. This pressure can harm the internal components of the cell and, in severe cases, increase the risk of thermal runaway.^[^ ^65^ ^]^ Zhang and their team investigated the decomposition of electrolytes and associated gas evolution issues arising when using propylene carbonate (PC) as a solvent in sodium‐based electrolytes. They demonstrated that sodium ions readily undergo ion–solvent complexation with PC, forming Na–PC complexes that significantly alter the electronic structure of the solvent. Due to this complexation, the Na–PC species exhibit a lower lowest unoccupied molecular orbital (LUMO) energy compared to pure propylene carbonate, as confirmed by first‐principles calculations. This reduction in LUMO energy enhances the likelihood of electrochemical reduction of the complexes on the sodium metal surface, leading to accelerated electrolyte decomposition, gas evolution, and the formation of multiple side products during the early stages of (SEI) formation.^[^ ^67^ ^]^

## 3 Dendrite Formation Theory

**Figure 6:**
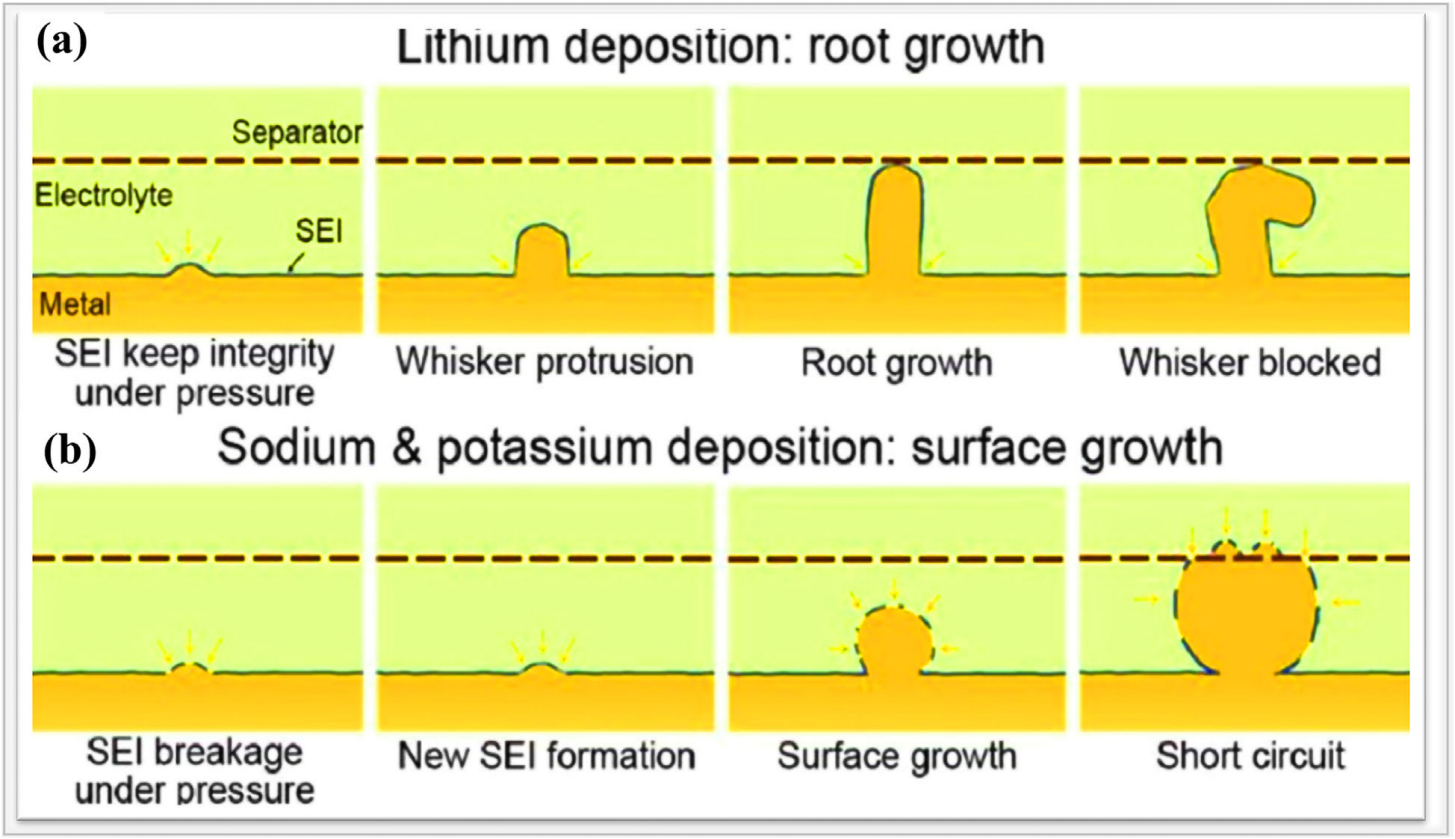
Illustration of interactions of lithium, sodium, and potassium with a Celgard separator depicting dendrite growth theories, including a) root growth mechanisms of Li and b) surface growth mechanisms for sodium and potassium. Reproduced with permission.^[^ ^176^ ^]^ Copyright 2021, Elsevier.

To reduce the occurrence of dendrites on metal anodes, it is crucial to first understand the mechanisms and theory behind their formation.^[^ ^68^, ^69^ ^]^ Feiyu Kang and their team investigated the deposition mechanisms of alkali metal anodes, including lithium, sodium, and potassium. The non‐uniform deposition of metal ions leads to protrusions that, after continuous cycling, result in capacity fading, low Coulombic efficiency, and short‐circuit issues, which urgently need to be solved. According to the deposition theory, three‐protrusion models have been proposed, namely, which are tip growth, root growth, and surface growth. Dendrite formation occurs at the tip, when the current density exceeds the diffusion rate, leading to rapid ion deposition. During this process, at the Sand's time defined above, the salt concentration near the electrode surface drops to zero, leaving the remaining ions to deposit unevenly on the surface protrusions. This results in the preferential growth of protrusions, as the scarce cations are drawn toward the tips of these structures. Tip growth is particularly dangerous compared to other modes, because the dendrites grow vertically rather than spreading across the surface. Also, they can pierce the separator, leading to internal short circuits and posing significant safety risks, including thermal runaway in extreme cases.^[^ ^70^, ^71^ ^]^ According to root growth theory, when a lithium metal battery operates below the limiting current density, the deposition process becomes inhomogeneous, owing to the heterogeneity of the SEI. As the deposition proceeds, pressure gradually accumulates beneath the SEI layer, causing it to stretch and eventually reach its mechanical limits. Beyond this point, the metal is pushed outward, forming protrusions. These whisker‐like structures continue to grow outward from the base under the tension created by the SEI, but their size is greater than nanopores, so they are blocked by the micropores of the separator. As a result, root growth is generally safer in terms of avoiding short circuits compared to other deposition modes. Surface growth occurs during cycling when there is a competition between SEI layer formation and lithium ions eposition, with the outcome being largely dependent on the current density. If the SEI layer forms before significant lithium‐ion deposition, it develops as a dense and uniform structure, and deposition tends to follow the root growth model. However, if lithium deposition begins first, the SEI may break apart and dissolve into the electrolyte.^[^ ^72^, ^73^, ^74^ ^]^ Then, deposition occurs directly on the lithium metal surface. That inhomogeneity spreads across the nanopores of the separator and causes short circuits. Then root growth mechanism occurs on lithium metal anodes, while the surface growth mechanism occurs on sodium and potassium metal anodes at a current density of 1 mA cm^−2^, as shown in **Figure** **6**a,b. During the deposition process in the case of lithium metal anodes, initially, the deposited lithium ions form an embryo through nucleation beneath the SEI layer. The formed SEI maintains its structural stability and continues to apply stress to the nucleated lithium. When cycling proceeds, the deposited lithium experiences continuous pressure. After some time, the lithium metal is squeezed out, and whisker‐type dendrites start growing from the root. However, these whiskers are typically larger than the nanopores of the separator, so they can't enter the separator and are effectively blocked, preventing short circuits. But in the case of sodium and potassium, the surface growth mechanism is applicable. During the deposition process, the SEI layer formed on the Na/K metal is not stable, so it cannot tolerate the pressure associated with deposition. At some point, the SEI breaks into pieces, and based on electron transfer reactions, a new SEI forms on the fresh metal surface. This deposition process includes three key steps: nucleation, SEI layer delamination, and new SEI layer formation. As this continues, granular‐shaped tiny dendrites grow without proper orientation on the surface. Also, these micron‐sized granules of Na/K grow without proper orientation and extend toward the separator, but do not penetrate it. Instead, they spread along the surface of the separator and eventually reach the opposite electrode side, which is referred to as the surface growth mechanism.^[^ ^74^, ^176^ ^]^

## 4 Need for a Stable SEI Layer

To prevent dendrite formation on the anode surface, the development of a highly stable SEI layer, also referred to as a passivation film, is essential. In metal anodes, the SEI layer forms naturally, as a result of the spontaneous reaction between the metal and the electrolyte components, which is beneficial for battery operation. This layer is stable, ionically conductive, and blocks electron flow from the sodium metal to the electrolyte, therefore, it is referred to as a passivation layer.^[^ ^75^ ^]^ During the plating process, ions can diffuse inside the SEI structure and deposit beneath it, owing to the ionic conductivity of the naturally formed SEI layer. However, if the SEI structure is nonuniform or heterogeneous, the deposition also occurs in a nonuniform manner. Consequently, during the stripping process, ions may not be completely removed, and some can become trapped in the SEI layer, forming nucleation sites that severely reduce the Coulombic efficiency of the battery. Once dendrite growth begins, either from the SEI layer or from the root of the metal, it becomes difficult to stop. This is because a higher concentration at one location causes subsequent ions to deposit in the same area instead of on other flat surfaces. Initially, small, needle‐like dendrites are formed. Over time, these dendrites grow and connect, forming branched structures with tree or snowflake‐like morphologies.^[^ ^24^, ^46^, ^76^, ^77^ ^]^ To enable the use of metal batteries without dendrite formation, the chemical and electrochemical properties of the interphase layer must be studied in its pristine state. Several studies have been conducted to generate stable natural SEI films using various electrolytes, solvents, salts, and additives in electrolyte engineering. While these studies have obtained some positive results, they are less promising for practical applications. This is because electrolyte modification introduces several issues, such as 1) continuous consumption of additives, which alters the electrolyte concentrations 2) formation of additive‐rich SEI layers due to the high electrolyte concentrations, making these layers resistant to ion transport. Based on the above considerations, some researchers have proposed an alternative solution, involving the replacement of electrolyte engineering with artificial SEI layer engineering.

### 4.1 Artificial SEI Layer

**Figure 7:**
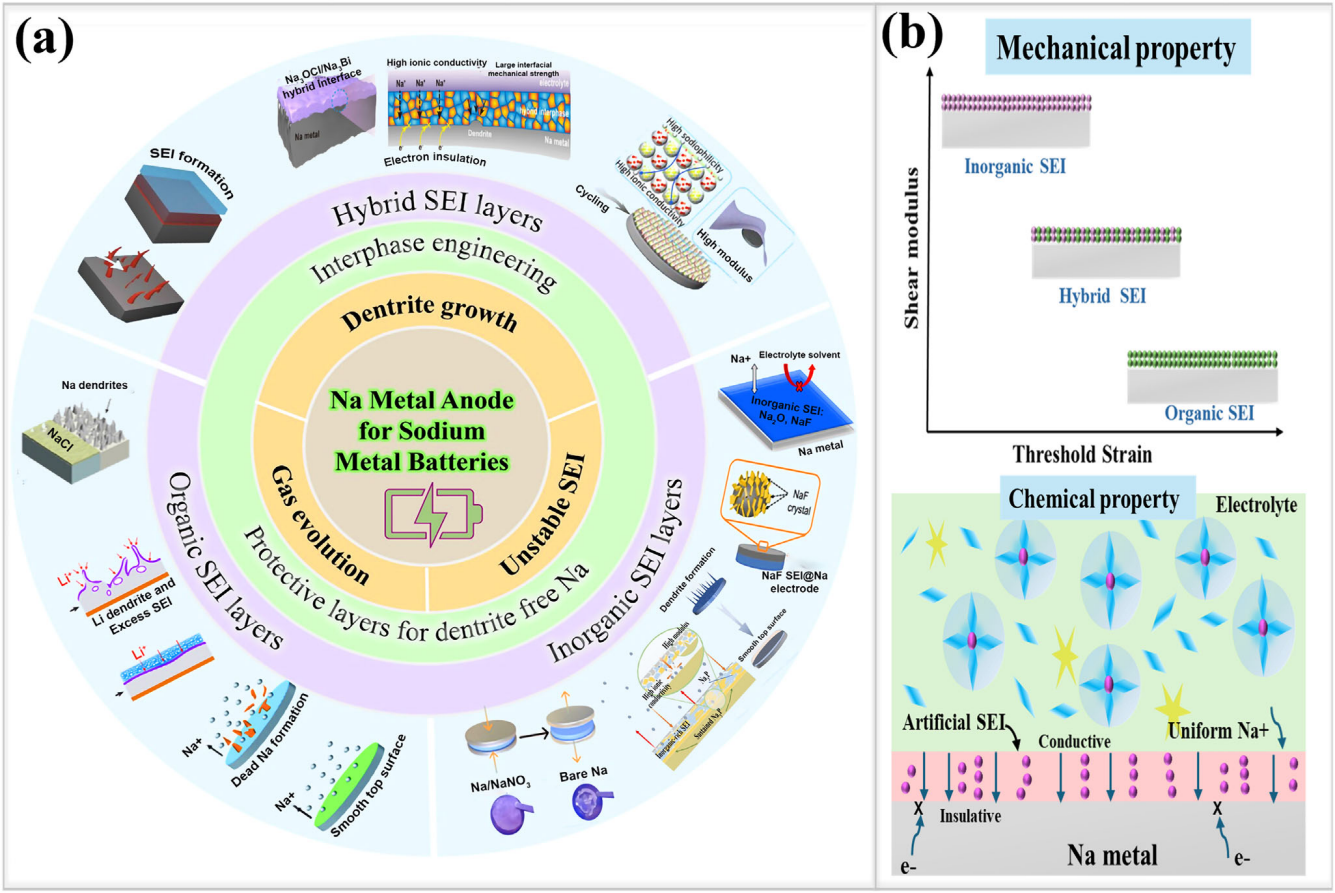
a) Illustration; Artificial SEI layers are classified into three types (organic, inorganic, and hybrid) based on their components. b) Typical characteristics of artificial SEI layers on alkali metal anodes; Key properties include 1) Mechanical strength, 2) Uniform Na^+^ deposition kinetics.

In this context, artificial SEI layers can play a crucial role in replacing the naturally occurring counterparts. These artificial layers must be stable enough to suppress aggressive dendrite formation. Theselected materials must be used to achieve this goal, with precise control over reaction time, chemical composition, thickness, and robustness. Also, such layers must have high Na^+^ conductivity, electron resistivity, and mechanical strength. Many types of artificial SEI layers are available, with superior chemical, electrochemical properties, and are designed effectively to address these challenges. The artificial SEI layer serves as a protective film that is ionically conductive, electrically resistant, and most importantly, prevents the contact between fresh sodium metal and the electrolyte once formed, thereby avoiding side reactions.^[^ ^78^, ^79^, ^80^, ^81^ ^]^ In the following sections, we discuss several types of artificial SEI layers, their formation methodologies, along their electrochemical performance. Based on their compositions, these SEI layers are classified into different types, namely, organic, inorganic, and hybrid layers, as shown in **Figure** **7**a,b.

### 4.2 Organic Interphase Layers

**Figure 8:**
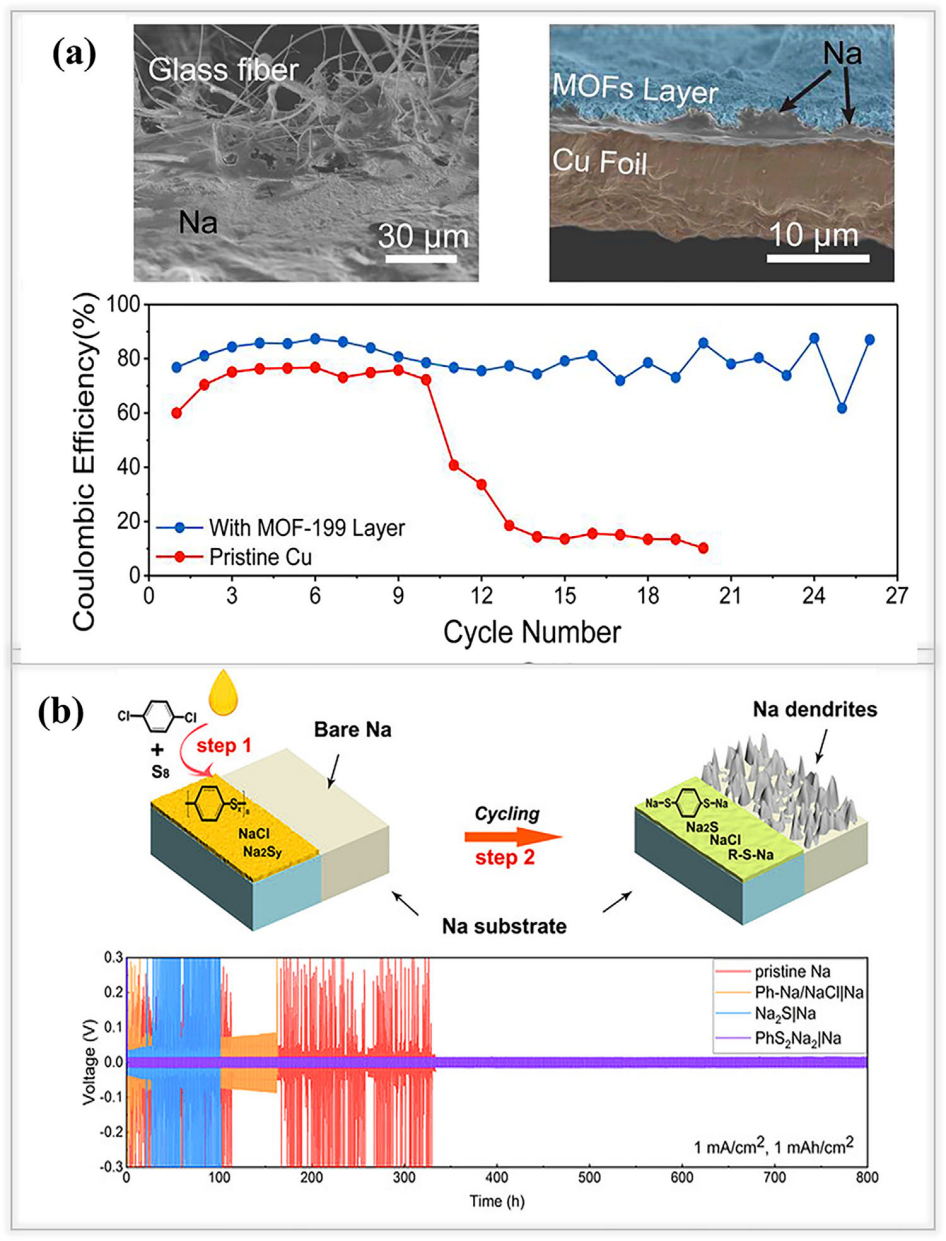
a) Metal–organic framework (MOF‐199) structures coated on Cu electrodes, comparing with pristine Cu foil. Top and cross‐sectional SEM images of pristine Cu and MOF‐199‐coated electrodes, after electrodeposition of 1 mAh cm^−2^ sodium. Electrochemical performance; Coulombic efficiency of pristine Cu and MOF‐199‐coated Cu electrodes at 1 mAh cm^−2^ and 1 mA cm^−2^.Reproduced with permission.^[^ ^82^ ^]^ Copyright 2019, Elsevier. b) Illustration of a sodium benzenedithiolate‐rich protective layer. Electrochemical performance; symmetric cells using pristine Na and PhS_2_Na_2_/Na at 1 mA cm^−2^ and 1 mAh cm^−2^. Reproduced with permission.^[^ ^83^ ^]^ Copyright 2020, Wiley‐ VCH.

Typically, organic SEI components effectively protect the sodium metal anode from dendrite formation, and as well as their elastic nature also avoids the volume expansion/contraction issues. Additionally, a low migration barrier promotes a more homogeneous Na^+^ deposition, enabling the production of dendrite‐free sodium metal batteries. Based on these criteria, metal organic framework (MOF)‐based artificial SEI layers, with high polarity, have been developed to protect both lithium and sodium, as recently reported by Qian et al. MOF‐199‐based artificial SEI layers provide some extra protection to sodium metal anodes via a liquid‐phase reaction method, as shown in **Figure** **8**a. After synthesizing the MOF networks, a slurry process was used to coat on Cu foil current collectors, followed by drying at 60 °C for 12 h. Cross‐sectional SEM images show that the deposited Na⁺ ions can penetrate the glass fiber separator, and as cycling proceeds, they tend to detach easily from the Cu surface. In contrast, the MOF‐199‐based coating adheres tightly to the Cu foil and acts as a stable protective layer, helping to regulate smooth and uniform sodium deposition during cycling. Subsequently, Na/Cu half‐cells were assembled to investigate the plating/stripping behavior of the MOF‐199‐based coating layer at a current density of 1 mA cm^−2^ and an areal capacity of 1 mAh cm^−2^. Without any protective layer, the pristine Cu electrode exhibited poor stability during the initial cycle, which deteriorated further in subsequent cycles. As a result, it achieved a low Coulombic efficiency of only 13.58%, after ten cycles. In contrast, the MOF‐199‐coated electrode demonstrated significantly enhanced stability and high Coulombic efficiency, highlighting the effectiveness of the protective layer in regulating sodium deposition.^[^ ^82^ ^]^

**Figure 9:**
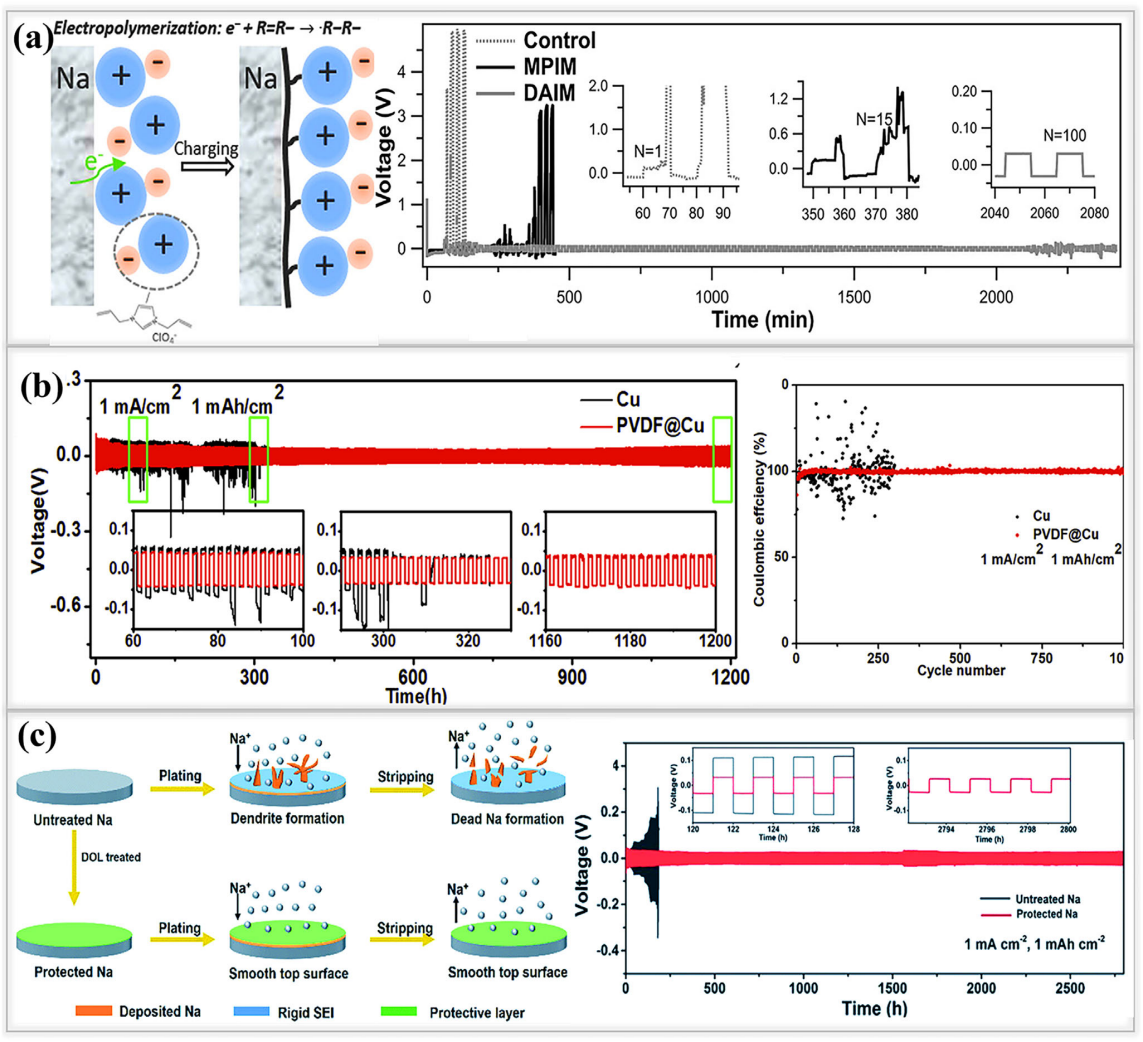
a) Scheme illustrating the formation of polymeric rich ionic membrane by electro‐polymerization reaction. Electrochemical performance of natural and artificial polymer membrane on sodium foil(Control, MPIM, DAIM): voltage versus time profiles under 1 mA cm^−2^ and 1 mAh cm^−2^ conditions: Reproduced with permission^[^ ^88^ ^]^ Copyright 2017, Wiley‐ VCH b) Plating/stripping behavior of PVDF polymer‐coated Cu cells and pristine Cu current collectors tested at 1 mAh cm^−2^ and 1 mA cm^−2^; half‐cell performance at 1 mAh cm^−2^ and 1 mA cm^−2^ demonstrating dendrite‐free characteristics. Reproduced with permission^[^ ^89^ ^]^ Copyright 2020, Elsevier c) Schematic representation of plating/stripping performance on untreated and protected Na; voltage versus time profile at 1 mA cm^−2^ and 1 mAh cm^−2^. Reproduced with permission^[^ ^90^ ^]^ Copyright 2021, RSC Publishing.

Wu and their team developed a sulfide‐rich organic interface layer (benzenedithiolate PhS_2_Na_2_) on sodium metal anodes through in situ reduction reaction, where a specific amount of para‐dichlorobenzene and sulfur (S₈) were dissolved in THF solvent. Then, 12 mm of sodium metal electrodes were dipped into the prepared poly (phenylene sulfide) (PPS) solution to form a sulfur‐rich polymer film on the surface. SEM images showed that the protective layer was ≈5 µm thick, effectively promoting even sodium ion deposition and significantly extending the plating/stripping lifespan to ≈800 h at 1 mA cm^−2^ 1 mA cm^−2^. To better understand how the PhS_2_Na_2_ protective layer interacts with sodium ions (Na⁺), a theoretical study was conducted and compared PhS_2_Na_2_ protected sodium SEI with commonly found SEI components, such as CH_3_ONa, CH_3_OCO_2_Na, and Na_2_CO_3_.Using density functional theory (DFT) calculations, found that PhS_2_Na_2_ had a significantly lower binding energy compared to the other traditional SEI components.^[^ ^83^ ^]^ This lower binding energy indicates higher ionic conductivity, allowing sodium ions to move more easily and deposit more efficiently beneath the SEI layers, as illustrated in Figure 8b. In recent years, electrochemical and polymerization reactions have emerged as promising strategies for forming a protective films by coating functional polymeric membranes onto metal anodes.^[^ ^84^ ^]^ Unlike nonaqueous electrolytes, these systems cannot be used directly in batteries and require pretreatment before being viable for battery applications, which complicates their large‐scale industrial use. To address this issue, ionic liquid monomers are introduced into the electrolytes, where they undergo chemical polymerization reactions, similar to how functional additives in electrolytes form in situ SEI layers on sodium metal anodes.^[^ ^84^, ^85^ ^]^ Using an electrochemical polymerization method, Wei and their team developed an ion‐rich polymeric membrane as an artificial SEI layer on sodium metal anodes, as shown in **Figure** **9**a.Ionic liquid monomers of the imidazolium cation type additives were introduced into the 1 m NaClO_4_ in EC/PC electrolyte. The schematic illustrates the polymerization process used to fabricate the ionic‐rich polymer membrane. The columbic efficiency study was investigated by following the Aurbach and group.^[^ ^86^, ^87^ ^]^ The optimized IL monomer‐based artificial SEI of (DAIM) achieved a high Coulombic efficiency of 95.0% at a current density of 1 mA cm^−2^, outperforming commercial electrolytes that either lack ionic liquid monomer additives (control electrolytes with no additives) or contain conventional monomers such as 1‐methyl‐3‐propylimidazolium chlorate (MPIM).The lower Coulombic efficiency (16.7%) of the control system is attributed to the high reactivity of sodium metal with inadequate aprotic liquid electrolytes. In the case of MPIM, which remains unpolymerized and was tested using the same procedure, a slightly better performance was observed compared to the control samples, but it was still ineffective in suppressing dendrite formation and achieving high Coulombic efficiency. In contrast, the DAIM (1,3‐diallyl imidazolium perchlorate)‐based SEI layer enhances sodium ion (Na⁺) reversibility due to the more uniform and ionically conductive polymeric film.^[^ ^88^ ^]^

Hou and their team prepared a polyvinylidene fluoride (PVDF) protective layer on a copper current collector using the doctor blade method to achieve dendrite‐free sodium ion deposition, as shown in Figure 9b. The PVDF@Cu layer contained SEI components such as Na_2_O_2_ and NaF, both possessing a high shear modulus and high ion migration. These properties not only suppress dendrite formation, but also facilitate smooth diffusion of sodium ions between the interphase. Thanks to its exceptional mechanical strength, the PVDF@Cu‐based SEI layer resulted in a longer lifespan of up to 1200 h with low overpotential(35 mV) in a symmetric cell at 1 mA cm^−2^ and 1 mAh cm^−2[^ ^89^ ^]^ Additionally, in a half‐cell configuration, the above layer achieved a Coulombic efficiency of 99.1% and a lifespan of 2000 h at 1 mA cm^−2^,1 mAh cm^−2^ and when the current density was increased to 2 mA cm^−2^, the cell achieved a 99.48% efficiency for 1000 cycles. Similarly, Mikhailova et al. introduced a protective layer on sodium metal anodes to address gas evolution and dendrite formation related issues, via a simple pretreatment method using 1,3‐dioxolane (DOL). The naturally formed SEI tends to be heterogeneous, leading to nonuniform sodium ion deposition during plating/stripping cycles. To improve the stability of the SEI layer, Mikhailova's team soaked the sodium electrodes in DOL solution for varying time zones of (1, 5, 10 min). This treatment triggered the polymerization of DOL, forming a uniform polymer layer on the sodium metal surface, as shown in Figure 9c. The polymer coating significantly enhanced the uniformity of the sodium ion deposition, reduced the charge transfer resistance, and drastically improved the lifespan of the system. The optimized system achieved an exceptional lifespan of 2800 h (1400 cycles) with a low overpotential of 25 mV at 1 mA cm^−2^ and 1 mAh cm^−2^.^[^ ^90^ ^]^

### 4.3 Inorganic SEI for Sodium Metal

**Figure 10:**
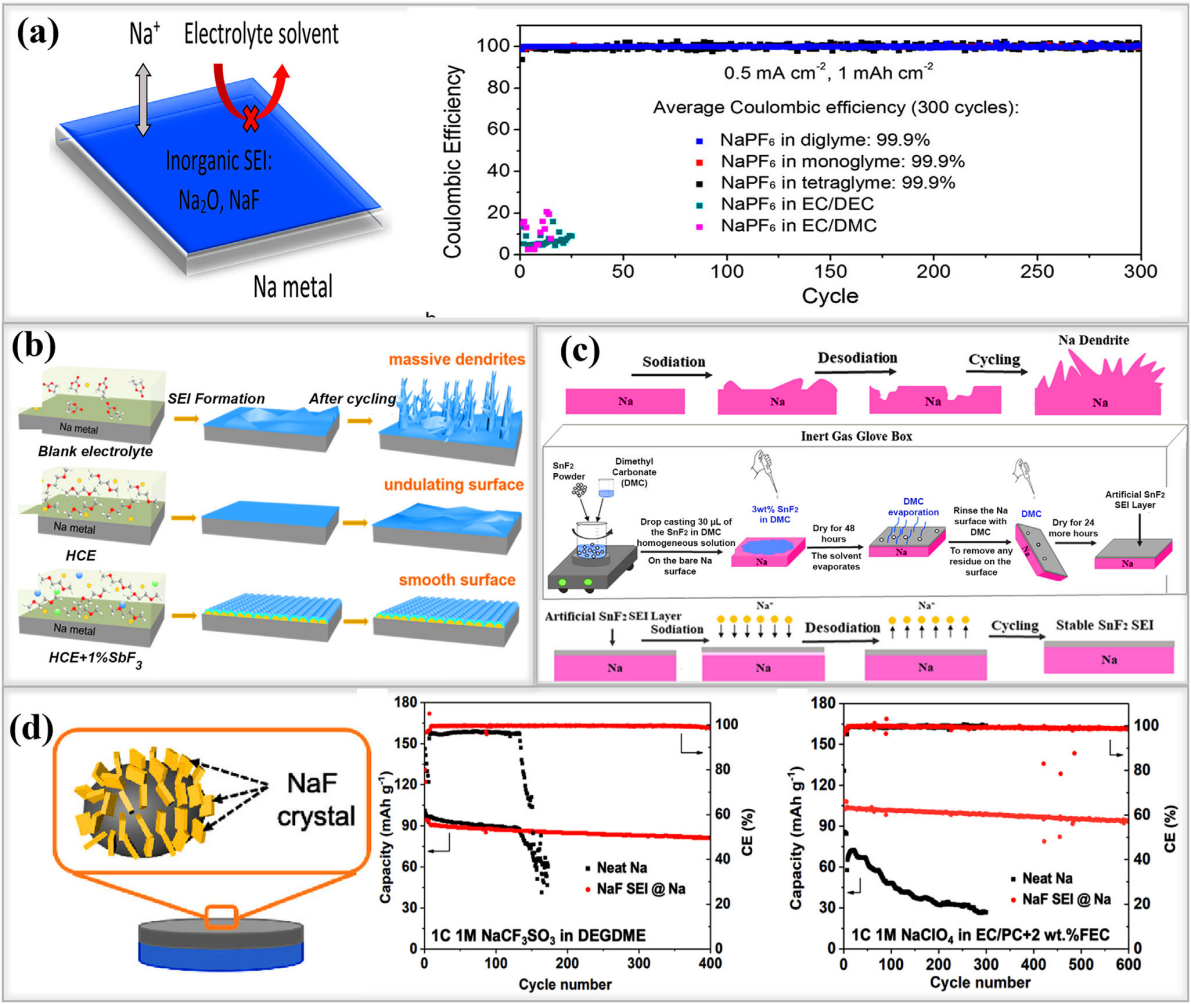
a) Schematic illustration of electrolyte reduction using (1 M NaPF_6_ in mono‐, di‐, and tetraglyme) on sodium metal foil. Coulombic efficiency measured for all variation of electrolytes at 0.5 mA cm^−2^ and 1 mAh cm^−2^. Reproduced with permission.^[^ ^28^ ^]^ Copyright 2015, American Chemical Society. b) Formation of conventional SEI layer from blank electrolyte, high‐concentration electrolyte, and high‐concentration electrolyte with 1% SbF_3_ additives. Reproduced with permission^[^ ^92^ ^]^ Copyright 2020, Elsevier c) Fabrication process of SnF_2_‐based artificial SEI layer on Na foil. Reproduced with permission.^[^ ^93^ ^]^ Copyright 2020, Elsevier d) NaF‐rich SEI on sodium metal anodes; electrochemical performances of NaF‐rich SEI and Neat Na evaluated in a full‐cell, using various electrolytes and along with NVP cathode. Reproduced with permission.^[^ ^94^ ^]^ Copyright 2022, Elsevier.

Various strategies have been developed to overcome the instability of the naturally formed SEI layer on sodium metal anodes, which is a key factor in limiting the cycle life of metal batteries. Among these approaches, inorganic‐based SEI layers are highly recommended for enhancing the longevity of sodium metal batteries. These layers provide high ionic conductivity, excellent mechanical strength, and uniform sodium ion deposition, effectively suppressing dendrite growth beneath the solid electrolyte interphase. Inspired by research on lithium metal batteries, where LiF‐based inorganic protective layers are widely used, a similar strategy has been applied to sodium metal anodes.^[^ ^11^, ^91^ ^]^ Using a NaPF_6_‐in‐glyme electrolyte (mono‐, di‐, and tetraglyme), Cui and his team successfully formed a compact inorganic SEI layer, composed of NaF and Na_2_O on sodium metal surfaces as shown in **Figure** **10**a, their findings suggest that thin, uniform SEI layer is highly compact and exhibits excellent mechanical strength, both of which are critical for suppressing dendrite formation^[^ ^28^ ^]^ Electrochemical performance tests using a half‐cell configuration with NaPF_6_‐in‐diglyme electrolyte showed that the cell achieved 300 dendrite‐free cycles with an electrochemical potential of (3.3–19.5 mV), along with an exceptionally high Coulombic efficiency of 99.9% at 0.5 mA cm^−2^, and 0.5 mA cm^−2^. Similarly, other electrolytes comprising 1 m NaPF_6_ in mono and tetraglyme solvents, electrochemical performance were also evaluated. Among these, the diglyme‐based electrolyte exhibited the highest Coulombic efficiency when compared to conventional carbonate‐based systems. Xiang and their team developed a bilayer protective coating composed of an inorganic‐rich NaF outer layer and a Na‐Sb inner layer. This structure was synthesized via a chemical treatment method involving SbF_3_ as an additive, dissolved in a high‐concentration electrolyte (HCE) formulated with 4 M NaFSI in DME. The prepared solution was drop‐cast onto the surface of the sodium metal anode, and visible color changes on the electrode surface confirmed the formation of the new SEI layer, as illustrated in Figure 10b. This bilayer configuration demonstrated exceptional interfacial stability, which was attributed to the synergistic interaction between the high‐concentration electrolyte and the SbF_3_ additive. It facilitated uniform sodium‐ion transport in symmetric cells for up to 1000 h at a current density of 0.5 mA cm^−2^.^[^ ^92^ ^]^ Lin and team designed a fluorine‐rich artificial SEI layer on sodium metal anodes by dissolving different weight percentages of tin fluoride (SnF_2_) in a DMC solution. This fluorinated protective layer is simple and inexpensive to prepare, making it a promising solution for achieving dendrite‐free sodium anodes with practical application in sodium metal batteries.^[^ ^93^ ^]^ Then 3 wt.% of the fluorinated SEI(SnF_2_/Na) layer ensured stable and uniform sodium ion transport, resulting in an extended lifespan of 700 h at 0.25 mA cm^−2^, 0.0625 mAh cm^−2^, with low overpotential, significantly outperforming pristine sodium metal anodes with less stable, naturally formed SEI layers, as shown in Figure 10c.

**Figure 11:**
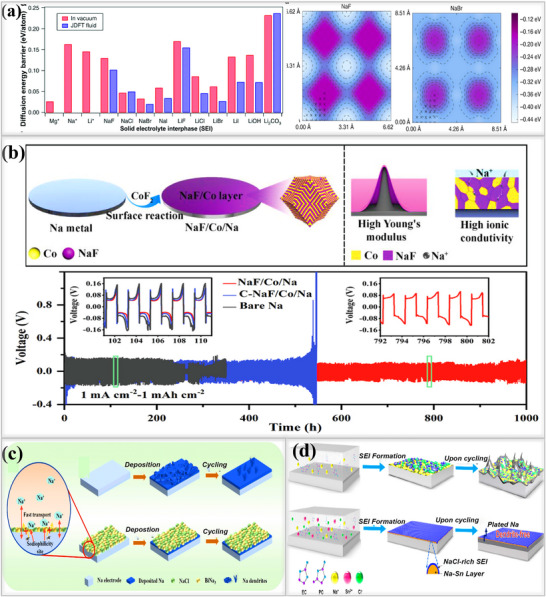
a) Theoretical results revealing surface diffusion barriers for Mg, Na, Li, and various SEI components: surface binding energies of NaF and NaBr sites calculated using joint density functional theory(JDFT).Reproduced with permission.^[^ ^95^ ^]^ Copyright 2017, nature communications. b) Schematic illustration of fabrication process for NaF/Co/Na interphases on sodium metal; voltage versus time profiles showing overpotential of bare Na and NaF/Co/Na in symmetric cells at 1 mA cm^−2^ and 1 mAh cm^−2^. Reproduced with permission.^[^ ^96^ ^]^ Copyright 2022, Wiley‐ VCH c) Illustration of cycling process for pristine Na and multiphase artificial SEI layer. Reproduced with permission^[^ ^97^ ^]^ Copyright 2024, RSC Publishing.d) Illustration of mosaic SEI formation on sodium metal anode using carbonate electrolyte, resulting in nonuniform deposition: NaSn and NaCl based interphases ensure uniform ion transport, preventing dendrite formation. Reproduced with permission.^[^ ^98^ ^]^ Copyright 2019, American Chemical Society.

Kim and their team designed a low‐cost, NaF‐rich SEI(NaF SEI@Na) layer by incorporating polytetrafluoroethylene (PTFE) micropowder into molten sodium at a 1:10 mass ratio. First, sodium metal was heated to 290 °C for 2 h in an inert atmosphere. Then, PTFE micropowder was added to the molten sodium, and the mixture was stirred slowly while being maintained at 290 °C for an additional 2 h, allowing the formation of a PTFE‐derived NaF‐coated SEI layer. The resulting NaF SEI@Na significantly improved electrochemical performance by suppressing dendrite growth and enhancing cycling stability.^[^ ^94^ ^]^ Full‐cell battery tests were conducted using both ether (1 m NaCF_3_SO_3_ in DEGDME) and carbonate‐based electrolytes(1 mM NaClO_4_ in EC/PC+2 wt.% FEC). In the ether‐based electrolyte, the cell delivered an initial capacity of 90 mAh g⁻¹ and demonstrated excellent cycling stability, retaining 92% of its capacity after 400 cycles at a 1C rate. In contrast, the carbonate‐based electrolyte enabled a higher initial capacity of 108 mAh g⁻¹ and maintained a capacity retention of 94% after 600 cycles, as shown. Figure 10d. The halide group NaX (X = F, Cl, Br, I) compounds have been applied for creating artificial SEI layers on sodium metal anodes. For example, Choudhury et al. used joint density functional theory to investigate the binding energy of sodium‐bonded halides based on their positions.^[^ ^95^ ^]^ In the case of the smallest halide, fluoride (F⁻), sodium binds directly on top of the fluoride ion in the most stable state, denoted as an anion site. Then, the position midway between two fluoride ions, called the in‐between site, has higher energy (less stable), which makes it difficult for sodium ions to move freely from the anion site to the in‐between site. As we move down the periodic table and the size of the halide anions increases(F⁻→Cl⁻→Br →I⁻), the binding energy of the in‐between site becomes closer to that of the anion site. In the NaBr system, the energy of the in‐between site is almost the same as that of the anion site, resulting in a very low energy barrier. This facilitates the easy movement of sodium ions from one site to another, enabling smooth diffusion and transportation. Their theoretical study revealed that NaBr, formed using pure sodium and 1‐bromopropane through the Wurtz reaction, exhibited a high ionic conductivity due to its low diffusion barrier. In addition, symmetric cells using NaBr‐coated electrodes demonstrated exceptional cycling stability for up to 250 h, maintaining a high Coulombic efficiency with minimal capacity fading as shown in **Figure** **11**a. Based on their findings, other research groups began exploring sodium halide‐based SEI layers

Yu et al. developed a heterogeneous NaF/Co interlayer, primarily composed of sodium fluoride (NaF) and metallic cobalt(Co). CoF_2_ nanoparticles were initially put onto the sodium metal anode and heated at 60 °C, where a replacement reaction occurred, leading to the formation of a heterogeneous protective layer on the sodium surface(NaF/Co/Na), as shown in Figure 11b. The proposed NaF/Co/Na‐based artificial SEI layer exhibited high ionic conductivity, high mechanical strength, and strong interfacial adhesion, helping to achieve a uniform sodium ion deposition.^[^ ^96^ ^]^ As expected, the resulting SEI layer significantly enhanced the cycling stability, reaching up to 1000 h at 1 mA cm^−2^ and 1 mAh cm^−2^, which is far superior to both commercially available CoF_2_‐coated electrodes and bare sodium metal anodes. Xie et al. developed a multi‐interphase layer on a sodium metal surface, incorporating a sodium‐rich Na_3_Bi alloy and NaCl to enhance the electrochemical performance of sodium metal batteries. This artificial SEI layer was prepared using a facile method, where BiCl_3_ was dissolved in THF solvent and then added dropwise onto the sodium metal surface, forming a multifunctional interphase layer. The high ionic conductivity and electron‐insulating properties of NaCl facilitate efficient sodium ion(Na^+^) diffusion within the SEI, while restricting electron tunneling from the metal to the electrolyte, effectively eliminating dendrite formation. Additionally, Na_3_Bi exhibits strong adsorption energy with sodium, ensuring a uniform ion deposition. To evaluate electrochemical performance, symmetric cells were assembled, incorporating the BiCl_3_/Na interphase under high current density (3 mA cm^−2^) and high areal capacity (3 mAh cm^−2^) conditions. These cells exhibited stable cycling performance for up to 1200 h, maintaining a flat voltage profile and a low overpotential of 20 mV. This stability was six times higher than that of pristine sodium, which lasted for only 190 h and exhibited an overpotential of 40 mV during initial cycles under the same conditions. This superior performance is attributed to the stable artificial SEI layer, which overcomes the sluggish sodium ion kinetics associated with natural SEI layers. DFT studies further confirmed the advantages of the multi‐interphase layer. Na_3_Bi was found to have the lowest adsorption energy with sodium atoms (−1.588 eV) along with a low nucleation barrier, making it more favorable for inclined deposition and enhanced ionic conductivity.^[^ ^97^ ^]^ Additionally, the sodium ion diffusion barrier of Na metal was calculated to be 0.423 eV, significantly higher than that of NaCl (0.315 eV). This suggests that sodium ions migrate more efficiently within the NaCl layer, contributing to the dendrite‐free deposition of sodium, as shown in Figure 11c. Huang and their team developed a stable and compact inorganic alloy interface, incorporating Na–Sn alloy and a NaCl‐rich interphase by dissolving SnCl_2_ additive in a carbonate‐based electrolyte(1 m NaPF_6_ in EC/PC or 1 m NaPF_6_ in EC/DEC).^[^ ^98^ ^]^ This approach effectively reduces the continuous electrolyte consumption, suppresses dendrite growth, and lowers the impedance, enabling the development of high‐performance dendrite‐free sodium metal batteries. The optimized 50 mm SnCl_2_ solution forms a stable protective interphase layer that strictly prevents direct contact between fresh sodium and the electrolyte, ensuring a uniform morphology. This engineered layer facilitates ultrafast Na^+^ transport between the interphase, maintaining stable cycling for up to 500 h at 0.5 mA cm^−2^, 1 mAh cm^−2^, a significant improvement compared to the blank electrolyte without additives, as shown in Figure 11d. Similar to the above report, another research group also utilized ZnCl_2_.^[^ ^99^ ^]^ and BiCl_3_^[^ ^100^ ^]^ to make a protective layer on sodium metal anodes.

**Figure 12:**
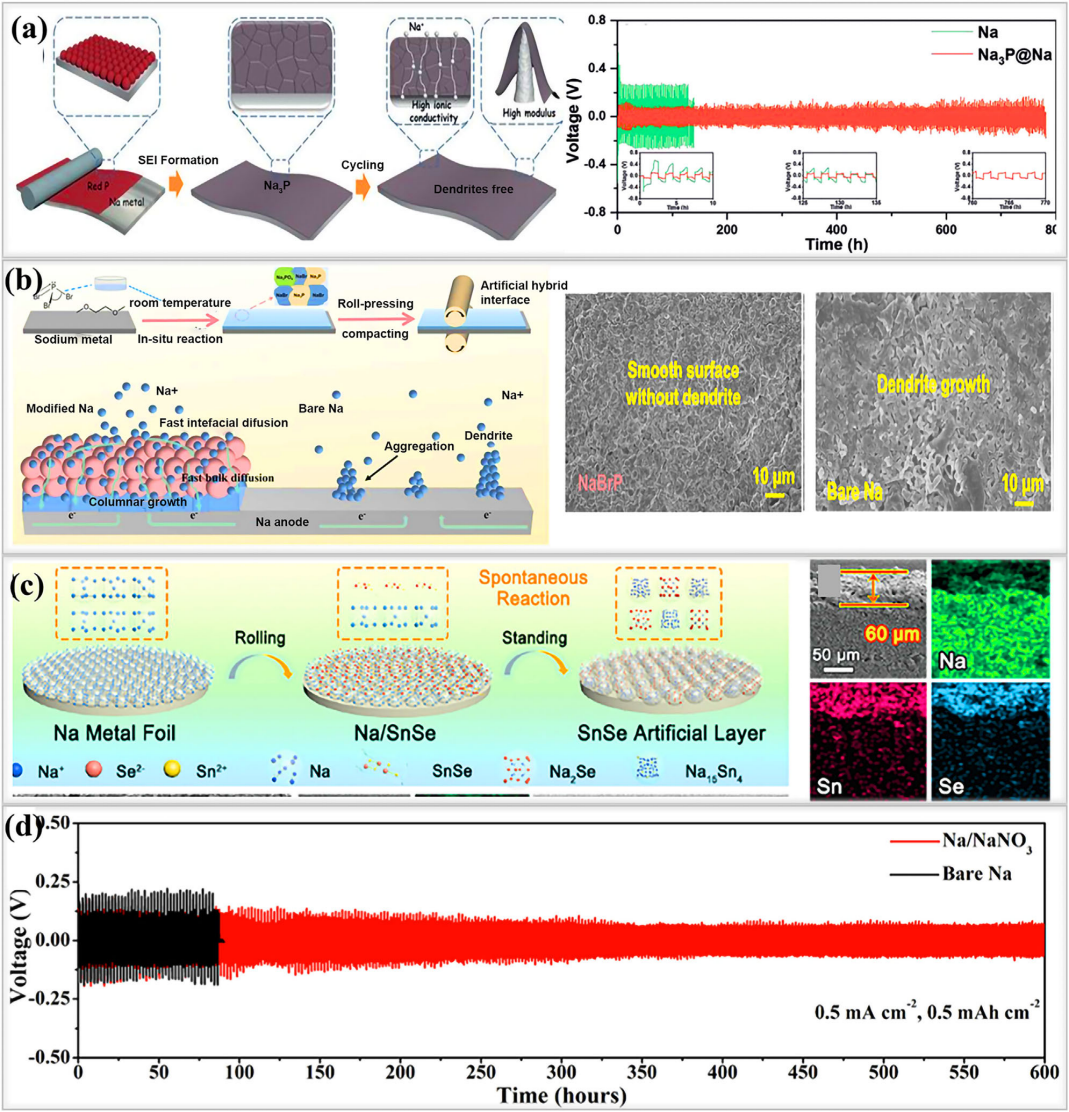
a) Fabrication process of Na_3_P layer on sodium metal surface. Electrochemical performance of symmetric cells using Na_3_P/Na anode versus pristine Na at low current density and capacity. Reproduced with permission.^[^ ^101^ ^]^ Copyright 2020, Wiley‐VCH. b) Schematic illustration of Na‐ion plating on NaBrP and bare Na surfaces: SEM images reveal that NaBrP coating prevents dendrite formation, whereas bare sodium metal experiences dendrite growth. Reproduced with permission.^[^ ^33^ ^]^ Copyright 2022, Elsevier. c) Illustration of fabrication process of Na/SnSe layer on sodium metal foil, along with cross‐sectional and EDS images confirming the new phase formation. Reproduced with permission^[^ ^102^ ^]^ Copyright 2024, Wiley‐VCH. d) cycling performance of Na/NaNO₃ and metallic Na at 0.5 mA cm^−2^ and 0.5 mAh cm^−2^. Reproduced with permission^[^ ^103^ ^]^ Copyright 2021, American Chemical Society.

In addition, Shi and their team developed an ex‐situ inorganic Na_3_P‐based interphase layer by applying red phosphorus powder onto sodium metal using a simple rolling method, as shown in **Figure** **12**a. This Na_3_P layer exhibited an ionic conductivity of 0.12 mS cm^−1^ and a low energy barrier of 11.0 kJ mol^−1^, which helped to maintain the stability of the artificial SEI^[^ ^101^ ^]^ Atomic force microscopy (AFM) experiments revealed that the Young's modulus of the Na_3_P layer was 8.6 GPa, significantly higher than that of pristine sodium (3.4 GPa). These superior electrochemical and mechanical properties promote uniform sodium ion flux within the SEI, reduce undesired side reactions, tolerate the stress and enhance the overall battery performance. Symmetric cells utilizing the Na_3_P interphase layer exhibited an exceptionally long lifespan of 780 h with a stable overpotential of 80 mV at 1 mA cm^−2^,1 mAh cm^−2^, demonstrating its effectiveness in regulating the interphase. This leads to a significantly improved cycle life compared to that of bare sodium, making the development of Na_3_P layer is a promising strategy for achieving dendrite‐free sodium metal batteries. Ji and their group rationally designed a robust artificial SEI(NaBrP) layer composed of Na_3_P and NaBr on the sodium metal surface.^[^ ^33^ ^]^ This layer forms through a spontaneous reaction between sodium metal and phosphorus tribromide (PBr_3_). Initially, PBr_3_ was dissolved in DME solvent, and the sodium metal was immersed in the solution, followed by a rolling process to compact the surface and enhance uniformity. The combination of Na_3_P and NaBr in the artificial SEI enhances the diffusion of sodium ions, eliminates dendrite formation, and mitigates the electrochemical challenges associated with sodium metal anodes. Cryo‐TEM images of the NaBrP layer revealed the presence of highly crystalline Na_3_P, NaBr, and Na_3_PO_4_ phases, providing sufficient pathways for fast sodium ion transport, as shown in Figure 12b. The interphase behavior was investigated by galvanostatically plating 5 mAh cm^−2^ of sodium ions onto both pristine and NaBrP‐coated surfaces. Aggressive dendrite formation, characterized by needle‐like structures and the presence of dead sodium, was observed on the pristine sodium surface, leading to the formation of a thick (≈86.5 µm) layer that blocked further deposition. In contrast, the highly conductive NaBrP layer facilitated compact sodium ion deposition beneath it, without dendrite formation. Then, symmetric cells utilizing the Na_3_P/NaBr protective layer exhibited a long lifespan of over 700 h (350 cycles) at 1 mA cm^−2^, 1 mAh cm^−2^, maintaining stable voltage profiles without disruptions. Geng and his team designed a multifunctional, highly sodio‐philic Na_2_Se/Na_15_Sn_4_ homogeneous structure through an in situ spontaneous reaction using sodium metal and SnSe nanoflakes by the rolling method at room temperature in a glove box.^[^ ^102^ ^]^ The interphase layer effectively bonded to the sodium metal and it was thermodynamically stable. SEM images revealed that the multifunctional Na_2_Se/Na_15_Sn_4_ SEI layer, exhibited high conductivity and enabled fast and uniform ions(Na^+^) transport across the interphase. Additionally, EDS images confirmed the successful formation of the SEI layer, while elemental mapping showed its uniform distribution within the interphase, as shown in Figure 12c. Owing to this in situ surface reconstruction, the SEI layer in symmetric cells exhibited an exceptionally long lifespan of over 900 h at 1 mA cm^−2^, 1 mAh cm^−2^, maintaining a flat voltage profile and a low impedance. In contrast, bare sodium cells failed after only 90 h, highlighting the effectiveness of the engineered SEI layer in enhancing the performance of sodium metal anodes. Sun et al. developed a Na/NaNO_3_ composite electrode using a mechanical kneading method to address the low solubility issues of the SEI layers.^[^ ^103^ ^]^ During this process, sodium metal foil and NaNO_3_ undergo continuous rolling/folding, forming a Na/NaNO_3_‐incorporated framework. The resulting ionically conductive composite foil, composed of NaN_x_O_y_ and Na_3_N SEI, had a thickness of 400 µm, facilitating rapid sodium ion transport Figure 12d. in particular, NaNO₃ was used as an SEI stabilizer and effectively addressed the solubility issues in the electrolyte. In addition to its high ionic conductivity, the composite foil exhibited enhanced mechanical strength, making it easier to cut into various shapes suitable for battery applications, compared to pristine sodium foil. The symmetric cells performance revealed that the Na/NaNO_3_ composite electrodes are stable until 600 h at 0.5 mA cm^−2^, 0.5 mAh cm^−2^, possessed significantly higher cycling stability than pristine sodium electrodes last only 90 h, which suffer from low solubility issues.

**Figure 13:**
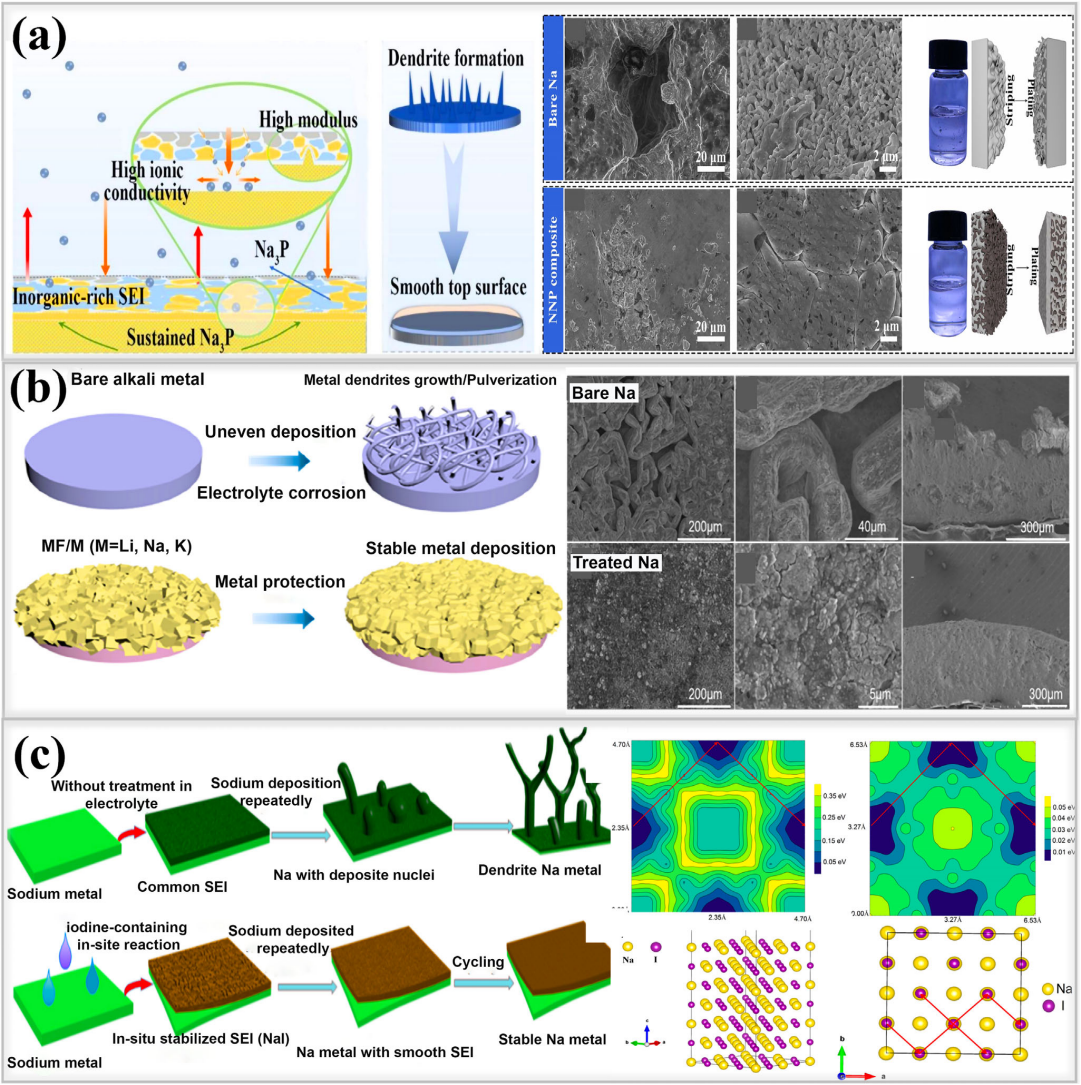
a) Schematic illustration of robust and ionically conductive inorganic‐rich SEI layer (NNP composite). Top‐view SEM images showing the morphology of bare Na and NNP electrodes after plating/stripping at 1 mA cm^−2^ and 1 mAh cm^−2^. After 50 cycles, both electrodes were dissolved in DMC solvent to observe their conditions. Reproduced with permission.^[^ ^104^ ^]^ Copyright 2024, Elsevier b) Illustration of fluorination reaction on alkali metal anodes, along with morphologies of bare Na after 30 cycles at 3 mA cm^−2^, including low, high, and cross‐sectional SEM images. Similar SEM images were obtained for Na/a‐SEI (treated Na) under the same conditions. Reproduced with permission^[^ ^32^ ^]^ Copyright 2022, American Chemical Society. c) Illustration of the formation of a stable NaI SEI layer on sodium metal, along with diffusion barrier on the surface investigated using density functional theory (DFT). Analysis of binding energy versus binding sites (Na adatoms on both NaF and NaI surfaces), also showing NaI slab and Na adatom pathways on NaI surfaces. Reproduced with permission^[^ ^38^ ^]^ Copyright 2019, Elsevier.

Sun and their group designed an inorganic‐rich Na/Na_3_P interphase with high ionic conductivity and high Young's modulus to facilitate smooth sodium deposition and ensure a uniform electric field distribution during the cycling process.^[^ ^104^ ^]^ A robust and conductive Na_3_P composite electrode was prepared using red phosphorus(P) and sodium metal(Na) through a mechanical folding, rolling method. The Na_3_P particles are uniformly distributed in the composite electrode, ensuring a consistent deposition behavior across the interface. In contrast, pristine sodium forms a fragile organic‐rich SEI layer, becomes unstable upon cycling. This instability leads to continuous dendrite growth, detachment of dead sodium, and potential dissolution into the electrolyte. The diffusion behavior after 50 cycles was investigated through SEM analysis, pristine sodium surface had an uneven appearance, with large grooves and cavities, indicating irregular ion deposition at 1 mA cm^−2^, 1 mAh cm^−2^. In contrast, the Na_3_P composite electrode exhibited a uniform surface, promoting rapid, uniform ion transport through multiple pathways. Additionally, both cycled electrodes were immersed in DMC solvent after 50 cycles to assess their stability. The pristine Na electrode showed numerous impurities, likely caused by inactive sodium, as shown in **Figure** **13**a. The high ionic conductivity of the Na_3_P interphase ensures clear solution without no impurity and improved performance in both symmetric and full‐cell systems, as demonstrated in this work. As shown in Figure 13b, Gu et al. designed a fluorine‐rich artificial SEI layer by simply dipping lithium or sodium into a mild organic fluorine reagent, triethylamine tri‐hydrofluoride (TREAT‐HF), dissolved in THF solvent.^[^ ^32^ ^]^ The reaction between the metal and the fluorine reagent forms a protective layer on the metal anode surface. The dipping time of the metal foil in solution directly influences the chemical composition, morphology, uniformity, and thickness of the interphase layer. SEM images of pure sodium and the fluorine‐coated Na/a‐SEI layer after cycling revealed distinct structural differences. The pristine sodium surface appears highly loose and structurally unstable, whereas the protective Na/a‐SEI layer is uniform and smooth. Additionally, the deposited sodium ions on the natural SEI layer form a relatively thick and nonuniform structure, primarily due to the heterogeneous nature of the SEI layer. In contrast, the deposited sodium beneath the fluorine‐coated SEI layer has a thin and smooth appearance, as the highly conductive Na/a‐SEI layer facilitates efficient sodium ion transport. Wang and colleagues created an ultrathin NaI layer on sodium metal anodes through the in situ reduction of 2‐iodopropane on sodium metal.^[^ ^38^ ^]^ The resulting NaI layer exhibited excellent cycling performance, achieving >2200 cycles at a 2C rate, a high capacity of up to 210 mAh g^−1^ at 0.5C rate. DFT calculations suggested that the ion diffusion barrier of the NaI layer was significantly lower (0.02 eV) than that of NaF(0.25 eV), which enhances the coulombic efficiency in sodium metal iodine batteries, as shown in Figure 13c.

Achieving a high electrochemical performance in sodium metal batteries critically depends on the thickness and uniformity of the SEI layer, which remains challenging to control at the metal electrolyte interface. Physical coating techniques represent a more effective approach for thickness control, providing greater uniformity and enabling the formation of highly stable SEI layers on sodium metal anodes. Moreover, in most studies, the interactions between organic or inorganic SEI components and the metal anode, including their composition and thickness, have not been thoroughly investigated. To address this gap, various physical deposition techniques have been proposed, in recent years for preparing artificial SEI layers on either current collectors or sodium metal anodes. These methods provide precise thickness control and are specifically designed to promote high uniformity in sodium ion transport near the interphase. Among these techniques, atomic layer deposition (ALD) is widely employed to create high‐quality protective layers with controlled thickness. Over the past several years, ALD techniques have been employed to form stable, thickness‐controlled protective layers on separators, cathodes, and anodes.^[^ ^105^, ^106^ ^]^ This kind of coating layer reduces the charge transfer resistance and enhances the electrochemical performance, making ALD is a promising approach for stabilizing reactive metal anodes.^[^ ^107^ ^]^

**Figure 14:**
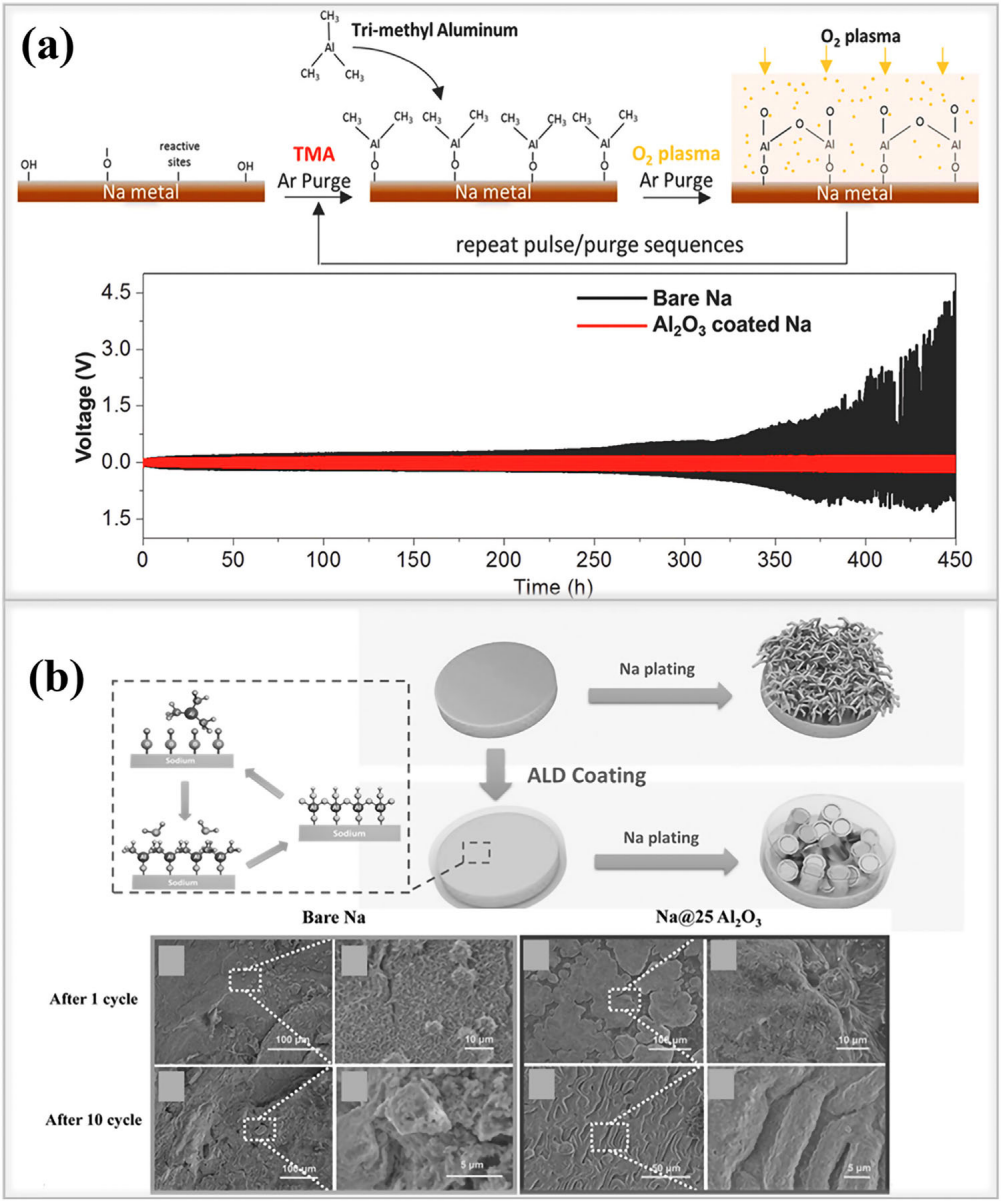
a) Schematic illustration of process involving 25 ALD cycles (2.8 nm) on sodium metal; symmetric cell performance tests for bare Na and ALD‐coated Na. Reproduced with permission^[^ ^108^ ^]^ Copyright 2016, Wiley‐ VCH b) Schematic comparison of sodium metal anodes with and without alumina oxide coating, as well as after plating; SEM images bare Na and Na‐Al_2_O_3_ (25 cycle‐coated) before and after cycles. Reproduced with permission^[^ ^109^ ^]^ Copyright 2017, Wiley‐ VCH.

As shown in **Figure** **14**a, Luo and his team developed an ultrathin(Al_2_O_3_)protective coating layer on the surface of a sodium metal anode using low‐temperature plasma‐enhanced atomic layer deposition (PEALD)for the first time.^[^ ^108^ ^]^ In the PEALD process, trimethylaluminum (TMA, serving as the aluminum precursor) and O_2_ plasma (used as the oxygen source) are introduced sequentially into the system. After each pulse, argon gas purging removes excess products and prevents side reactions, ensuring layer‐by‐layer deposition, as per the ALD principle. The growth rate is 1.1 Å per cycle, with 25 cycles producing a coating of ≈2.8 nm thickness. To address the high reactivity of sodium with air and moisture, the PEALD process was conducted inside a glove box under argon atmosphere. This precise thickness control at low temperatures resulted in a stable and uniform coating. X‐Ray photoelectron spectroscopy (XPS) analysis confirmed the successful deposition of Al_2_O_3_ on the sodium metal surface, without any side reactions. To evaluate electrochemical performance, symmetric cells were assembled and tested using both Al_2_O_3_‐coated Na and bare sodium, with 1 m NaClO_4_ in EC:DEC as the electrolyte. Both cells underwent 30 min of plating/stripping at 0.25 mA cm^−2,^ and the bare sodium cell exhibited a gradually increasing overpotential upon cycling, indicating that the naturally formed SEI layer in the carbonate‐based electrolytes was insufficient to prevent sodium metal corrosion. In contrast, the Al_2_O_3_‐coated sodium gives a low potential, achieving a lifespan of 450 h. This finding demonstrates that the uniform Al_2_O_3_ coating significantly facilitates the smooth deposition of sodium ions during the plating/stripping process, contributing to improving the battery stability and performance. However, focusing on the specific thickness of the Al_2_O_3_ coating cannot fully capture the advantages of ALD coatings on sodium metal. Further optimization is necessary to determine the ideal thickness for industrial applications. To address this issue, Sun and his team optimized interphase layers with various thicknesses and identified a 25‐cycle Al_2_O_3_ as the most effective layer for protecting sodium metal anodes from dendrites^[^ ^109^ ^]^ as shown in Figure 14b.To examine the impact of this ultrathin coating during cycling, SEM measurements were conducted before and after 10 cycles. The bare sodium sample exhibited significant morphological changes, including large grooves, mossy‐like dendrites observed after cycling. In contrast, the ALD‐coated sodium subjected to 25 cycles Al_2_O_3_ maintained an island‐like morphology, which remained consistent before and after cycling. Additionally, Rutherford backscattering spectrometry (RBS) experiments were performed to analyze the depth profile and elemental composition of both bare Na and Al_2_O_3_‐coated sodium samples before and after 50 cycles. Both samples exhibited S peaks, indicating electrolyte interactions with the surface during cycling. However, the presence of Al peaks in the ALD‐coated sodium sample confirmed that the aluminum layer remained intact even after cycling, maintaining a high yield percentage. The wider distribution of Al in the coated sample suggested a partial intermixing of the Al_2_O_3_ layer with Na–S–O phases. However, the areal density of sulfur in the ALD‐coated sample was significantly lower than that in bare sodium. This finding highlights the ability of the Al_2_O_3_ coating to suppress sulfur accumulation and minimize side reactions, ultimately contributing to improving the cycling stability.

**Figure 15:**
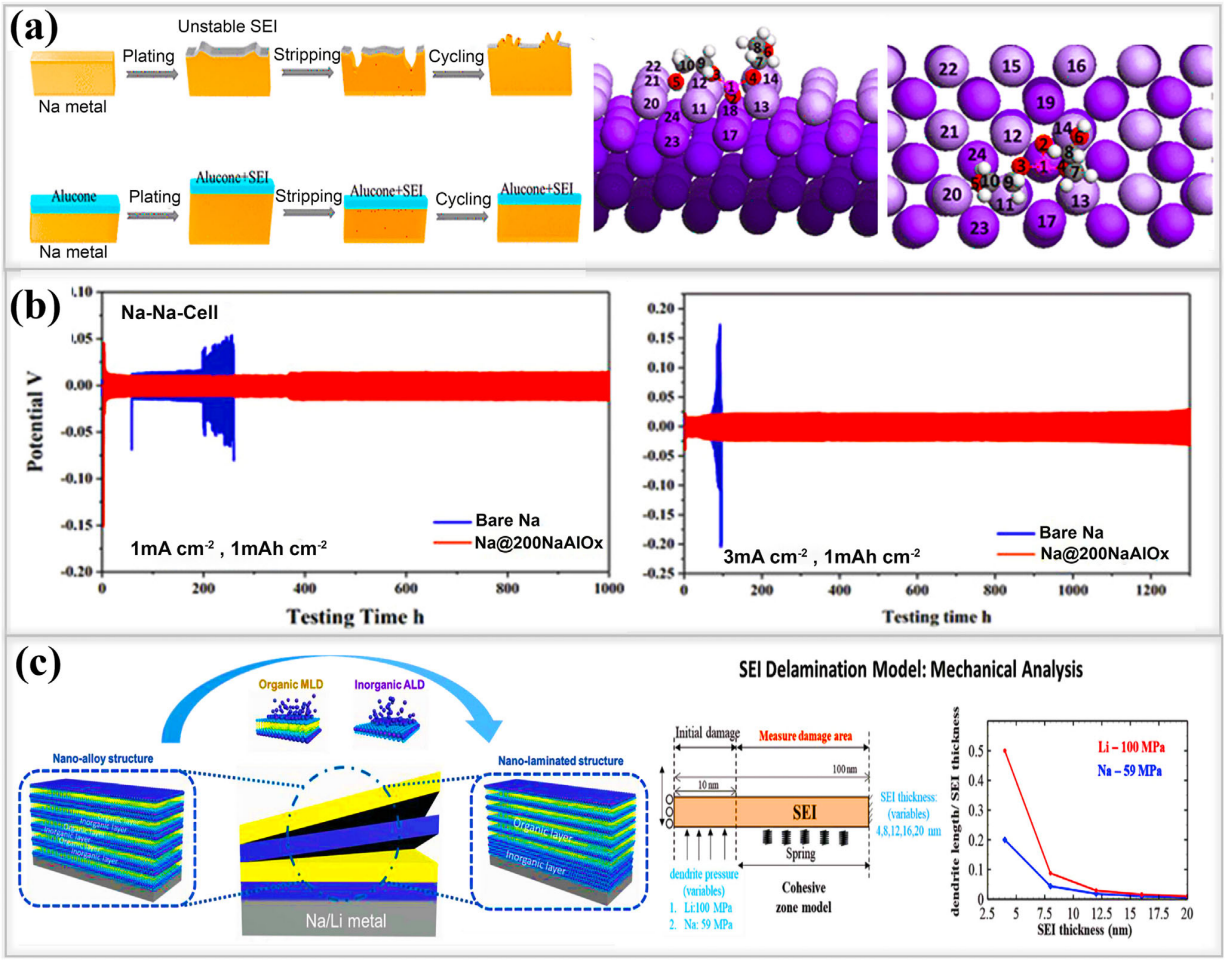
a) Illustration of MLD alucone coating on sodium foil, along with plating/stripping process. 3D view adsorption of single alucone molecule on Na (100) surface. Reproduced with permission.^[^ ^110^ ^]^ Copyright 2017, American Chemical Society. b) two‐step strategy to design an ionic conductive layer on Na metal anodes; electrochemical performance symmetric cell study, Na@200NaAlO_x_ anode and Bare Na, under various working conditions. Reproduced with permission^[^ ^111^ ^]^ Copyright 2022, Wiley‐ VCH. c) Illustration of the design of nanoalloy and nano‐laminated interfacial structures, along with SEI delamination analysis based on cohesive zone model. Reproduced with permission^[^ ^112^ ^]^ Copyright 2023, Wiley‐ VCH.

Molecular layer deposition (MLD) is a thin‐film deposition technique that provides precise control over the film thickness at the molecular level. Similar to ALD, MLD operates via a layer‐by‐layer deposition process, but also incorporates organic components as second precursors, alongside the inorganic precursors. This hybrid approach provides greater functionalization options, as the oxygen precursor in traditional ALD (O_2_, H_2_O) can be replaced with organic linker groups. Additionally, MLD is performed at relatively low temperatures, increasing its versatility. Sun and colleagues were the first team to successfully design and deposit a hybrid inorganic/organic protective alucone layer on sodium metal anodes using the MLD technology.^[^ ^110^ ^]^ In their process, TMA was used as the inorganic precursor, while ethylene glycol served as the organic precursor, and the deposition was carried out at 85 °C in an argon‐filled glove box, as shown in **Figure** **15**a. The resulting alucone layer on sodium metal was also analyzed theoretically, using DFT and ab initio molecular dynamics simulations. These analyses revealed strong interactions between the alucone layer and sodium metal, primarily attributed to Na─O and Na─Al bonds. Such interactions stabilize the sodium surface, effectively preventing corrosion from electrolytes and suppressing sodium dendrite growth. To optimize the thickness of the alucone coating, MLD was performed for 10, 25, and 40 cycles. Among the obtained samples, sodium metal coated through 25 MLD cycles (denoted as Na@25 Alucone) exhibited the best performance, maintaining a flat voltage profile and stable plating/stripping behavior for 270 h at a current density of 1 mA cm^−2^ and a capacity of 1 mAh cm^−2^, significantly outperforming bare sodium metal(160 h). For comparison, Sun's team also prepared Al_2_O_3_ coatings on sodium metal by ALD, using TMA and water as precursors. Electrochemical tests comparing the ALD and MLD coatings revealed that Na@25 Alucone exhibited superior performance under various working conditions. This improvement is attributed to the protective effect of the alucone layer, which prevents the direct sodium/electrolyte contact while providing high mechanical strength. Before and after cycling, the Na⁺ deposition morphology in bare Na and Na@25 alucone layers was investigated using SEM analysis. Bare sodium (Na) metal forms 3D sphere‐like dendrites, and after cycling, this increases the surface area and leads to poor efficiency. However, coating Na with an alucone layer results in a much smoother surface with fewer dendrites. This smooth surface ensures uniform Na deposition and improves battery performance. Therefore, the alucone layer effectively prevents harmful dendrite growth and enhances efficiency.

Sun's team further extended their investigations to 1) ALD methods for forming lithiated layers on lithium metal or sodiated layer on sodium metal, and 2) the effect of the deposition temperature and the composition of the SEI on both lithium and sodium metal.^[^ ^111^ ^]^ For this purpose, they employed a two‐step strategy, by carefully controlling the ALD process temperature of both below the melting point of lithium (120 °C) and close to its melting point (120–180 °C). In the case of the Na metal anode, the maintained temperature is either below the sodium melting point (65 °C) or close to it (97.8 °C). In this approach, ALD is carried out at a low temperature, followed by a post‐treatment step where the temperature is increased. This strategy enabled the formation of artificial lithiated (Li‐based) and sodiated (Na‐based) SEI films without requiring additional lithium or sodium sources. These artificially constructed SEI layers exhibited excellent ionic conductivity and delivered outstanding electrochemical performance in both symmetric (Li/Li, Na/Na) and full (Li and Na) cells, as shown in Figure 15b. Further investigations revealed that the temperature plays a crucial role in the ALD process. For instance, Li@LiAlO_x_ coatings produced below the melting point of lithium formed uniform and smooth surfaces. However, when the temperature exceeded the melting point, the coatings became rough and mechanically unstable after cycling, likely due to thermal effects impacting the lithium metal surface. In summary, MLD and ALD technologies represent promising solutions for enhancing the stability and performance of alkali metal anodes. The precise control over the thin film thickness, the ability to create hybrid organic/inorganic layers, and temperature optimization are key factors for developing advanced protective coatings.

Zhao and his team developed organic/inorganic hybrid layers ranging from nanoalloy to nano‐laminated structures using a combined ALD/MLD process for Li and Na metal anodes, as shown in Figure 15c. They demonstrated the effectiveness of hybrid coatings (ALD‐Al_2_O_3_, MLD‐alucone) and also investigated the mechanical properties of the SEI layer using a cohesive zone model (CZM).^[^ ^112^ ^]^ To identify the optimal thickness of a unit structure, they tested three ALD/MLD cycle variations: (1 layer ALD:1 layer MLD), (2 layer ALD:2 layer MLD), and (5 layer ALD:5 MLD). Each variation involved different deposition cycles(5,10,25), in which the symmetric cell based on Na@ (1 ALD:1 MLD)10, with an Al_2_O_3_ alucone hybrid layer, exhibited excellent cycling stability, reaching 1500 h at a current density of 3 mA cm⁻^2^ and a capacity of 1 mAh cm^−2^. Using cohesive zone modeling (CZM), the authors elucidated why lithium requires a thicker SEI compared to sodium. The simulations considered an initial damage zone, representing regions where the SEI is fully delaminated due to outward mechanical pressure from Li/Na dendrite nucleation. The stress distribution and resulting dendrite lengths were evaluated for SEI thicknesses ranging from 4 to 20 nm. Because lithium has a smaller molar volume than sodium, the mechanical stress induced during its electrodeposition (100 MPa) is significantly higher than that of sodium (59 MPa). At a thickness of 4 nm, the SEI on Li fails to suppress dendrite growth due to the high stress, while Na remains stable under the same conditions. Therefore, the study concludes that lithium requires a thicker SEI layer to ensure mechanical integrity and effective dendrite suppression. Under typical conditions, a 20 nm SEI is considered optimal for Li, though higher deposition pressures may necessitate even greater thicknesses for long‐term stability. Furthermore, ALD provides highly controlled SEI layers, but is significantly more expensive than chemical‐based approaches. This presents a trade‐off, where researchers must balance costs and ease of preparation, especially given the challenges in controlling the composition of the SEI layer.^[^ ^16^ ^]^ To achieve a high electrochemical performance, cost factors must be carefully taken into account to determine the feasibility of these techniques for commercial applications.

### 4.4 Hybrid Interphases

As mentioned above, artificial SEI layers effectively suppress dendrite growth, volume expansion, gas evolution, and unwanted side reactions, making them suitable for real‐time applications. In particular, while organic SEI layers provide flexibility, their mechanical strength and ionic mobility are relatively poor. On the other hand, inorganic SEI layers provide high ionic conductivity and satisfactory interphase strength, helping to mitigate sluggish ion kinetics issues. However, not all organic and inorganic phases meet these requirements or ensure a long cycle life. To achieve a high battery lifespan, a combination of both organic and inorganic SEI layers is employed to enhance the overall properties, including ionic conductivity, mechanical strength, and flexibility of the sodium metal anode. This synergistic approach significantly improves electrochemical performance and is far more effective than studying the SEI layers separately. The integration of hybrid SEI layers also represents a promising direction for advancing sodium metal battery technologies.^[^ ^113^ ^]^

**Figure 16:**
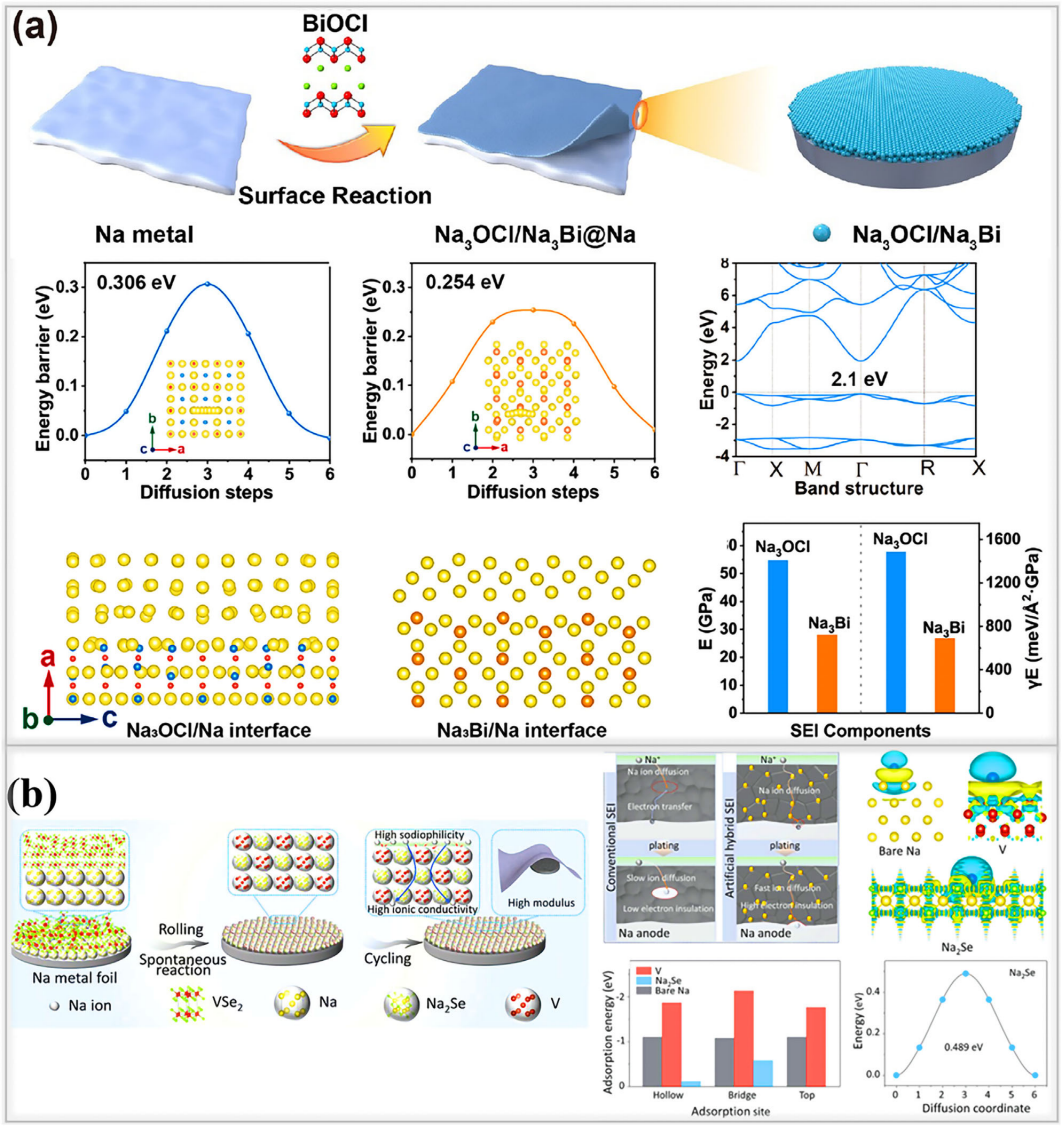
a) Schematic representation of synthesis procedure for hybrid Na_3_OCI/Na_3_Bi@Na interphases. This hybrid SEI layer exhibits high ionic conductivity and excellent mechanical strength. A theoretical study provided insights into the energy barrier for Na‐ion transport in the Na_3_OCI/Na_3_Bi@Na hybrid phase. Band gaps and interfacial energy analysis using DFT calculations. Reproduced with permission^[^ ^114^ ^]^ Copyright 2022, American Chemical Society. b) Illustration of design process of heterogeneous Na_2_Se/V interphase and its advantages, along with evaluation of diffusion mechanism for conventional and ideal SEI, as well as corresponding adsorption energies and charge densities. Reproduced with permission^[^ ^115^ ^]^ Copyright 2022, Wiley‐ VCH.

Yu's research group has actively researched sodium metal anodes to achieve dendrite‐free sodium metal batteries.^[^ ^114^ ^]^ Their rationally designed hybrid SEI layer consists of alloy (Na_3_Bi) and solid electrolytes (Na_3_OCl) formed by reducing an oxyhalogenide on sodium metal at room temperature, as shown in **Figure** **16**a. The resulting hybrid interphase provides multiple pathways for uniform deposition of sodium ions, because both Na_3_Bi and Na_3_OCl are ionic conductors. Additionally, Young's modulus of the hybrid layer (9.4 GPa) is three times higher than that of bare sodium(2.8 Gpa), significantly improving the mechanical stability of the interphase. Theoretical studies were conducted to further investigate the exceptional properties of this hybrid SEI layer. Both ionic conductors exhibited a low migration barrier, indicating enhanced ionic diffusion. The band gap of Na_3_OCl was determined to be 2.1 eV, suggesting that the solid electrolyte component effectively restricts electron tunneling from the metal anode to the SEI, allowing only ions to deposit beneath the SEI layer. In contrast, Na_3_Bi is semi‐metallic in nature(0.13 eV), contributing to the electronic insulation of the interphase. The combination of high Young's modulus and strong interfacial energy ensures surface stability, uniform ion distribution, and electronic insulation, ultimately enhancing the cycle life and preventing aggressive dendrite formation. Yu's research group introduced a multifunctional interphase layer using disodium selenide (Na_2_Se) and vanadium (V) on sodium metal foil, via a simple mechanical roll‐pressing method to achieve the Na@Na_2_Se/V interphase layer.^[^ ^115^ ^]^ The resulting hybrid layer exhibited an exceptionally high lifespan of 1790 h in symmetric cells at 0.5 mA cm^−2^, 1 mAh cm^−2^, while full‐cell tests showed a stable performance even after 1800 cycles at a 5C rate. To better understand the deposition behavior of this layer, the conventional SEI model was developed in this work. The artificial layers were formed in a controlled manner and exhibited a sturdy composition, enhancing rapid ion transport and ensuring the deposition of sodium ions beneath the interphase. In contrast, the conventional SEI layer exhibited poor diffusion rates and weak electronic insulation, causing sodium ions to deposit inside the SEI rather than beneath it. To further investigate the superior properties of the artificial SEI layer, DFT calculations were conducted to calculate the adsorption energy across three different models. The results suggested that the adsorption energy of Na^+^ on V(all hollow, top, and bridge sites) is negative, and lower than that of other variants such as Na_2_Se, bare Na, indicating strong interactions involving sodium ions and metal vanadium. Additionally, the charge density difference analysis revealed a high affinity of V toward Na ions compared to other variants, while the calculated energy barrier for Na_2_Se was significantly lower, contributing to a high ionic conductivity as shown in Figure 16b.

**Figure 17:**
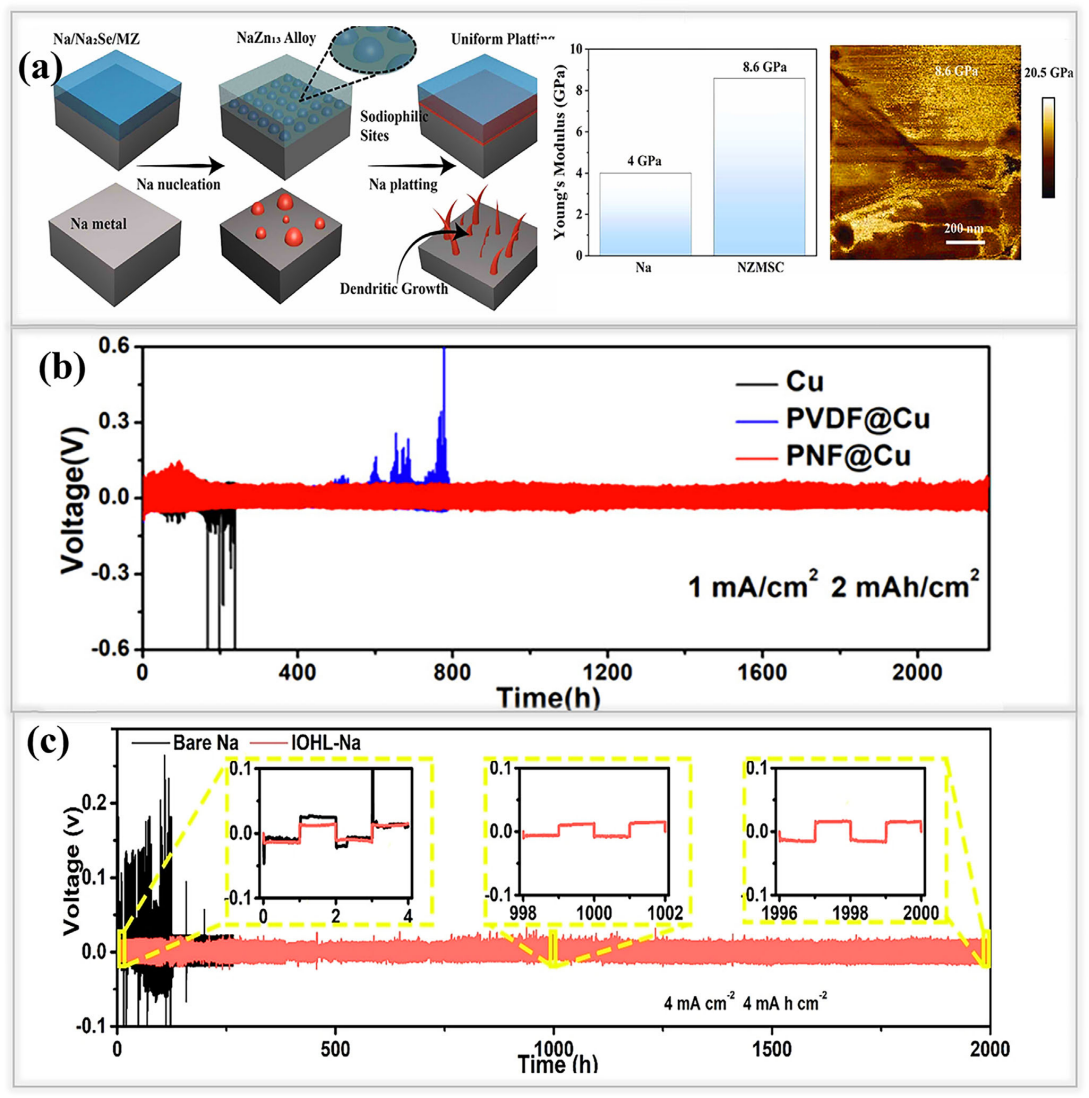
a) Illustration of Na‐ion transport on bare Na and NaZn₁₃ alloy; Young's modulus distribution of bare Na and Na/Na₂Se/MZ. Reproduced with permission^[^ ^116^ ^]^ Copyright 2024, Elsevier. b) hybrid protective layer and bare Cu foil; symmetric cell performance of respective electrodes. Reproduced with permission^[^ ^117^ ^]^ Copyright 2019,American Chemical Society. c) Symmetric cell performance of bare Na and IOHL‐Na at 4 mA cm^−2^,4 mAh cm^−2^. Reproduced with permission^[^ ^118^ ^]^ Copyright 2019, American Chemical Society.

Yousaf et al. developed a multicomponent hybrid artificial SEI layer (Na_2_Se/MZ) composed of Na_2_Se, Mn, and Zn metals through a chemical reaction induced a rolling and folding procedure, which conducted in an argon‐filled glovebox.^[^ ^116^ ^]^ According to DFT results, the Na_2_Se/MZphase is ionically conductive, Mn enhances the interphase strength, and the NaZn_13_ alloy provides abundant sodio‐philic sites, facilitating efficient transport of sodium ions. During the initial cycles, single Zn metal atoms transform into the NaZn_13_ alloy, creating active deposition sites, ensuring a smooth sodium ion transport across the SEI layer. In comparison, bare sodium exhibits a high nucleation and as well as growth potential, which promotes dendrite growth on its surface, leading to severe stability issues. The mechanical strength of the hybrid SEI layer was evaluated through AFM experiments, the young's modulus of Na_2_Se/MZ(8.6 GPa) was twice that of bare sodium (4.0 GPa), demonstrating the superior mechanical stability of the hybrid layer as shown in **Figure** **17**a. Quan et al. designed a hybrid protective layer on a copper foil current collector using PVDF and NaF via the blade coating method.^[^ ^117^ ^]^ The successful coating of the artificial SEI layer (PNF@Cu) and, for comparison, PVDF@Cu was verified using X‐ray diffraction (XRD) and XPS measurements. SEM images revealed smooth surface morphology, while EDS analysis indicated that both NaF and PVDF particles were uniformly mixed and coated on the surface of the current collectors. Additionally, symmetric cell tests were conducted for each electrode to evaluate their plating/stripping performance at 1 mA cm^−2^ and 2 mAh cm^−2^ areal capacity. The PNF@Cu electrodes exhibited an exceptionally long lifetime of 2100 h, as shown in Figure 17b. Jiao et al. developed a multifunctional hybrid SEI layer (IOHL‐Na) using SnCl_2_ and 4‐chloro‐2,6‐dimethylphenol solution, which was dropped on sodium metal anodes Figure 17c. The integrated inorganic and organic components of the SEI layer provide a high Young's modulus and strong interactions, while the organic SEI components of aromatic polymers introduce an elastic structure, enhancing the electrochemical performance.^[^ ^118^ ^]^ The half‐cell performance was evaluated in an asymmetric setup to measure the Coulombic efficiency during the cycling. The results showed that IOHL‐Na achieved a Coulombic efficiency of 98.5% over 800 cycles at 2 mA cm^−2^, 2 mAh cm^−2^, and their voltage profile indicates that IOHL‐Na significantly reduces the electrolyte consumption and ensuring a stable battery system. In contrast, bare sodium exhibited a highly fluctuating Coulombic efficiency, which was difficult to measure due to uncontrolled parasitic reactions. Additionally, symmetric cells with hybrid interphase integration exhibited stable cycling performance up to 2000 h, with a low potential of 15.8 mV at 4 mA cm^−2^, 4 mAh cm^−2^

**Figure 18:**
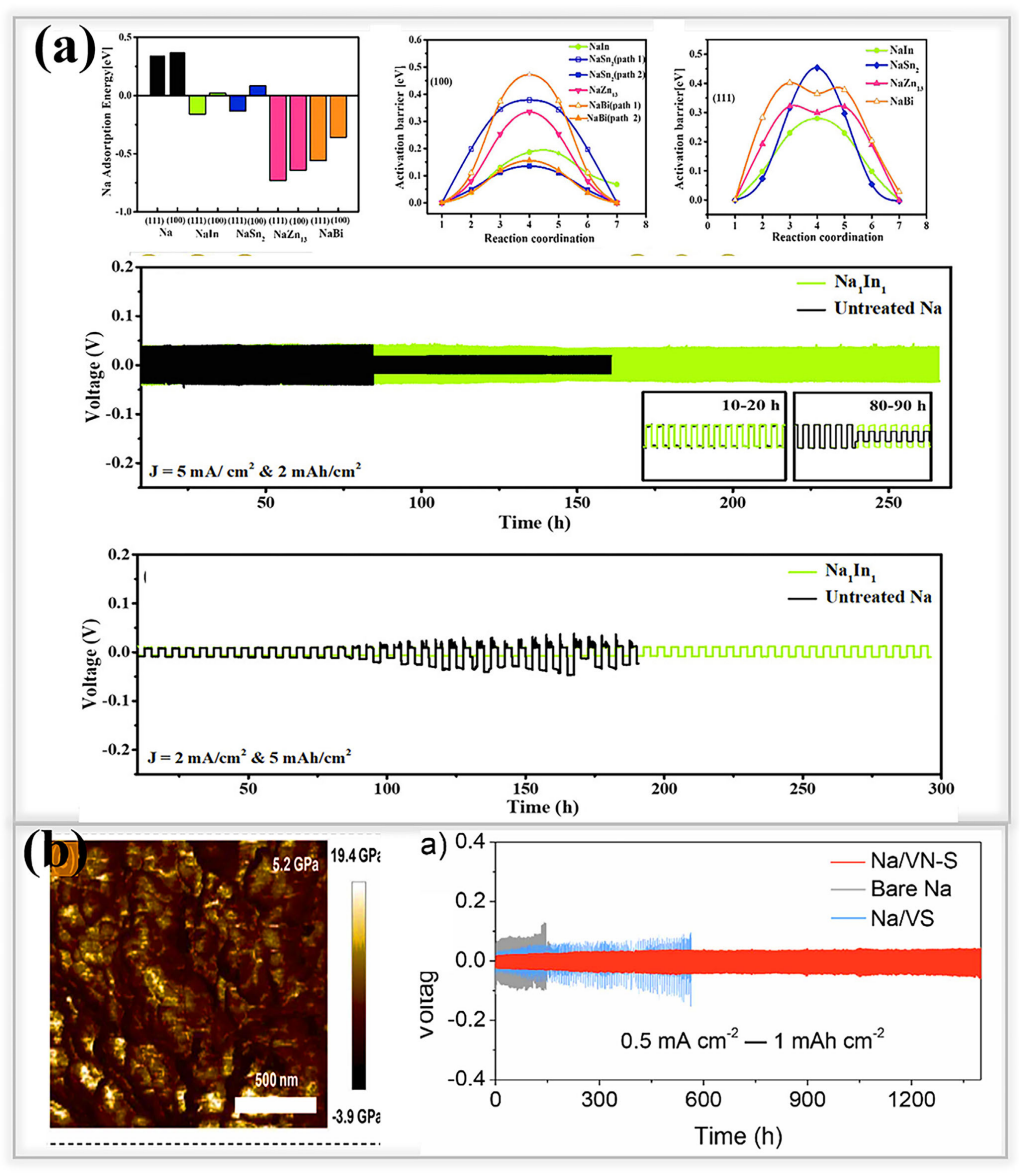
a) Adsorption energy barriers and surface energies (DFT) for several sodium‐ion conducting interphases, including Na–In, Na–Sn, and Na–Bi,Na‐Zn; cycling stability tests conducted at high areal capacity and high current density. Reproduced with permission^[^ ^119^ ^]^ Copyright 2023, Wiley‐VCH b) Young's modulus analysis for hybrid Na/VN‐S system and symmetric cell performance. Reproduced with permission^[^ ^120^ ^]^ Copyright 2024, Elsevier.

**Figure 19:**
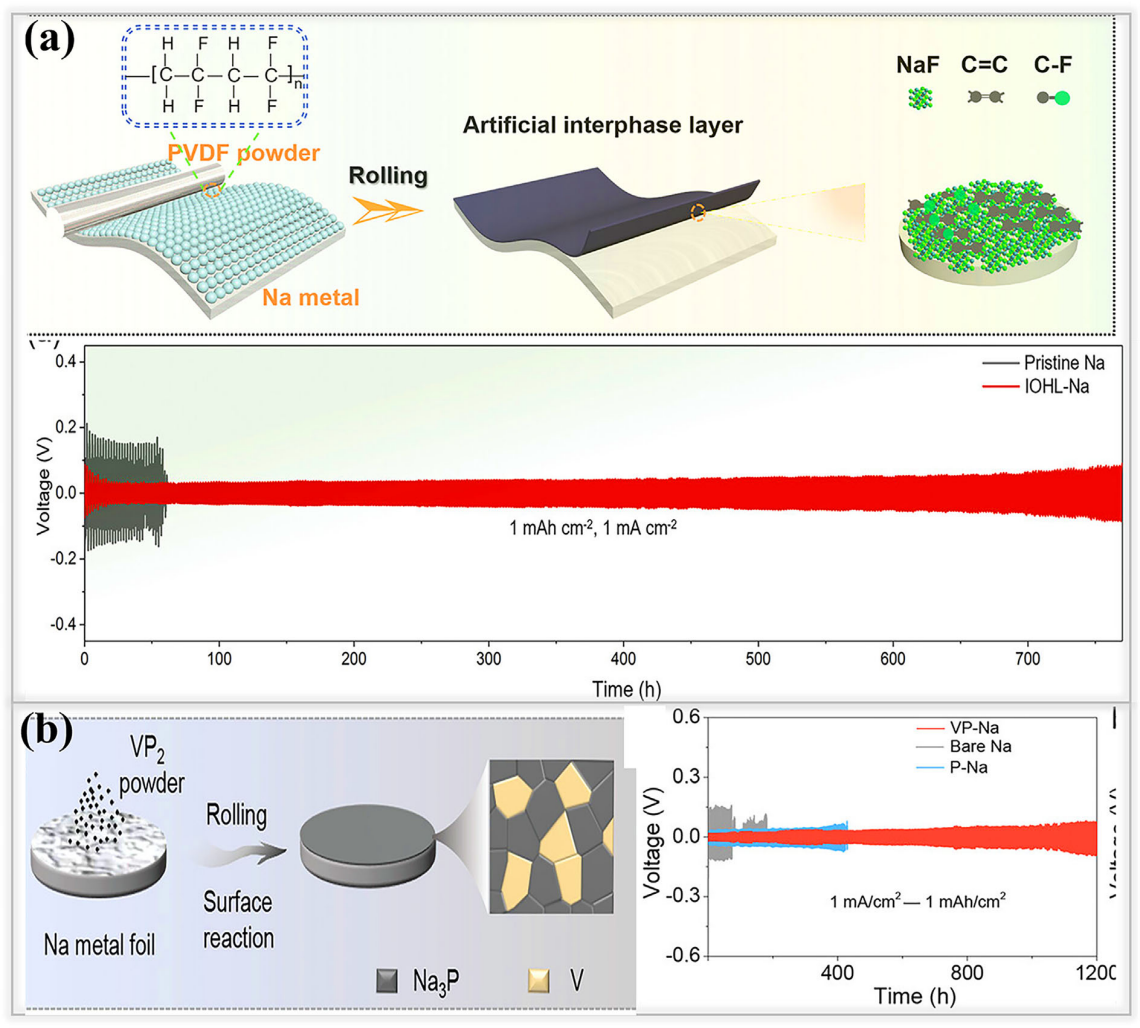
a) Schematic illustration of the preparation of IOHL‐Na; symmetric cell performances of pristine Na and IOHL‐Na. Reproduced with permission^[^ ^121^ ^]^ Copyright 2023, Wiley‐ VCH. b) Illustration of synthetic process of artificial hybrid interphase layer(VP‐Na);^[^ ^122^ ^]^ Copyright 2023, Wiley‐ VCH.

Lee et al. investigated a series of sodium‐ion‐conducting hybrid interphases, including Na–In, Na–Bi, Na–Sn, and Na–Zn alloys on sodium metal anodes via chemical reactions using the drop‐casting method **Figure** **18**a. DFT analyses revealed that low diffusion barriers and surface energies are the key factors facilitating uniform sodium ion deposition near the metal anode.^[^ ^119^ ^]^ The hybrid SEI layers exhibited stable behavior even at high current densities (5 mA cm^−2^, 2 mAh cm^−2^) and areal capacities(5 mAh cm^−2^, 2 mA cm^−2^). The as developed SEI layers howed high ionic conductivity, helping to achieve Na^+^ deposition beneath the SEI, and maintained excellent cycling performance under extreme working conditions compared to pristine Na. Their structural robustness enables the SEI layers to withstand sudden pressure and stress, ensuring a good cycling stability throughout prolonged battery operation. Xia et al. rationally designed a hybrid artificial SEI layer (Na/VN‐S) using vanadium nitride(VN), metallic vanadium(V), and sodium sulfide (Na_2_S) through a chemical pretreatment process.^[^ ^120^ ^]^ The high sodio‐philicity, mechanical strength, low adsorption energy, and low nucleation barrier of the Na/VN‐S system make it a unique structure for realizing dendrite‐free sodium and a lifespan up to 1400 h at 0.5 mA cm^−2^ and 1 mAh cm^−2^. Then, the hybrid SEI layer and bare sodium morphology during plating were analyzed using optical microscopy. In Na/VN‐S system, uniform plating was observed on the sodium surface without any noticeable dendrites, primarily due to the low ion migration barrier, which prevents nucleation inside the SEI and facilitates uniform deposition beneath it. In contrast, bare sodium showed visible formation of dendrites within 10 min of plating, which continued to evolve into small and eventually tree‐like or mossy dendrites. These structures have a high risk of piercing the separator, leading to battery failure Figure 18b. Yu and his team developed a hybrid interphase layer (IOHL‐Na) containing inorganic/organic components (NaF/C–F/C═C) on the surface of sodium metal foil using PVDF and metallic sodium through an in situ reaction **Figure** **19**a. The hybrid SEI protective layer was created by rolling PVDF powder with sodium foil inside a glove box, leading to two key reactions: defluorination (inorganic NaF formation) and dehydrogenation (organic C═C, C─F bonds) of PVDF on sodium metal.^[^ ^121^ ^]^ This integrated structure enhances the ionic conductivity, sodio‐philicity, mechanical strength, and surface compatibility, significantly improving the sodium ion kinetics and overcoming the corresponding slow transport issues. The successful coating of the artificial hybrid SEI layer on sodium metal foil was further confirmed through XPS measurements, in which F1s and C1s spectra indicated the occurrence of PVDF defluorination and dehydrogenation reactions. To evaluate the electrochemical performance of the hybrid layer, symmetric cells were assembled with IOHL‐Na‐based sodium electrodes. The results showed a stable cycling for up to 770 h, with negligible voltage hysteresis at 1 mA cm^−2^ and 1 mAh cm^−2^, demonstrating a good reaction kinetics even during continuous plating/stripping in carbonate electrolytes. In contrast, bare sodium exhibited poor cycling performance and rapid degradation under the same conditions. The authors also evaluated the full‐cell performance using IOHL‐Na as the anode and Na_3_V_2_(PO_4_)_3_ (NVP) as the cathode, demonstrating an excellent rate performance across a wide range of C‐rates (from 1C to 25C). In comparison, bare sodium failed to withstand higher current densities, leading to early cell failure. The IOHL‐Na electrodes maintained an efficient sodium‐ion transfer even under high‐rate conditions, making them highly promising for sodium metal battery applications. Furthermore, the researchers explored ultra‐low (−40 °C) and ultrahigh (55 °C) temperature conditions to evaluate the thermal stability of the hybrid SEI‐coated sodium anodes. Symmetric and full‐cell tests conducted at these extreme temperatures revealed stable capacity retention and consistent ion transport behavior, confirming that temperature variations did not affect the interphase compatibility, sodio‐philicity, or mechanical strength.

Additionally, Rui et al. developed another hybrid SEI layer (VP‐Na) containing sodium phosphide (Na_3_P) and metallic vanadium via a chemical pretreatment process, as shown in Figure 19b. Owing to its exceptional mechanical strength and low activation energy barrier, the VP‐Na hybrid SEI layer facilitated rapid sodium ion transport and effectively suppressed dendrite formation.^[^ ^122^ ^]^ SEM analysis was performed to investigate the sodium deposition behavior after cycling. The VP‐Na hybrid SEI layer exhibited smooth and uniform morphology, attributed to its structural stability and strong interphase composition. In contrast, bare sodium showed dead Na accumulation and dendrite growth, leading to poor cycling performance. Further symmetric and full‐cell tests confirmed that the VP‐Na hybrid SEI layer maintained stable operation across a wide temperature window, ranging from ultralow to ultrahigh conditions. These results suggest that the developed hybrid SEI layers not only provide high cycling stability at room temperature (similar to previous studies), but also remain stable under extreme temperatures, making them promising anode materials for high‐energy‐density sodium metal batteries.

## 5 Understanding Deposition Behavior Using In Situ Tools

For the successful application of metallic sodium anodes in next‐generation high‐energy‐density batteries, researchers must thoroughly understand their interfacial chemistry, Na^+^ deposition behavior, and interconnections between them. This knowledge is crucial for overcoming associated challenges and advancing the commercialization of sodium metal batteries. Therefore, alongside investigations into metal anodes, equal emphasis should be placed on precise characterization using advanced analytical tools to enhance research in this field.^[^ ^123^ ^]^ A few ex situ studies have been conducted to investigate the SEI layer formation, composition, and uniformity. The key techniques employed in these studies include XRD, to study the crystal structure of the artificial SEI layer,^[^ ^124^, ^125^ ^]^ SEM, to obtain insights into the morphology of sodium, XPS,^[^ ^126^, ^127^, ^128^ ^]^ to identify the chemical composition of the SEI layer before and after cycling. Fourier transform infrared (FTIR) spectroscopy, to analyze the chemical components of the SEI layer,^[^ ^62^, ^129^, ^130^ ^]^ time‐of‐flight secondary ion microscopy(TOF‐SIMS),^[^ ^131^, ^132^ ^]^ a highly sensitive surface analysis method to investigate both sodium metal and its interphase by sputtering the sample with either argon or gallium ion sources. This technique provides 2D/3D surface images to explore the uniformity of the SEI components and also performs depth profiling.^[^ ^39^, ^133^ ^]^ electrochemical impedance spectroscopy (EIS),^[^ ^134^ ^]^ to evaluate the charge transfer resistance between the metal and electrolyte interphase. AFM, which provides topographical images and insights into mechanical properties.^[^ ^135^ ^]^ However, studying the various properties of the SEI layers before and after assembly often requires disassembling the cell, which also poses significant challenges due to the high reactivity of sodium with the atmosphere. This reactivity can alter or damage the physical, chemical, electrochemical, electrical, and mechanical properties of the SEI layers, making it difficult to obtain accurate information about the battery. To address these issues, it is crucial to develop in situ methods to analyze the surface chemistry of real sodium metal anodes. Several advanced in situ tools have already been developed for lithium/sodium metal anodes and are considered promising analytical techniques for studying the deposition behavior, nucleation, growth, and dendrite formation during the plating/stripping process.^[^ ^43^, ^123^, ^136^ ^]^ In the following sections, we describe the principles of some advanced in situ tools and summarize their real‐time applications in detail, focusing on recent advances in this field.

### 5.1 In Situ Optical Microscopy

**Figure 20:**
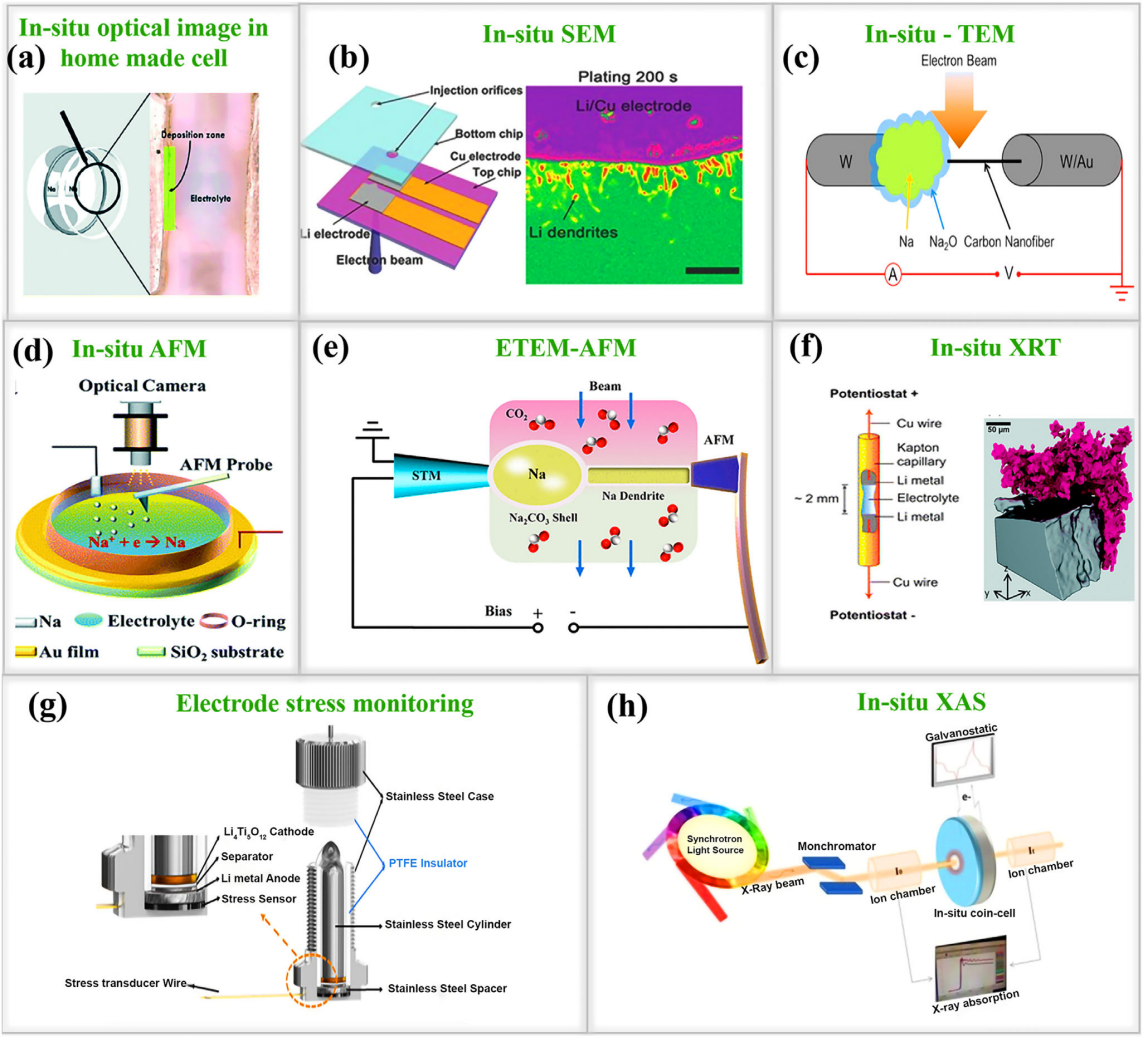
In situ characterization tools used to analyze sodium metal anodes: a) In situ optical images of homemade cells. Reproduced with permission^[^ ^137^ ^]^ Copyright 2017, American Chemical Society. b) In situ SEM Reproduced with permission^[^ ^139^ ^]^ Copyright 2017, Wiley‐ VCH c) In situ TEM Reproduced with permission^[^ ^142^ ^]^ Copyright 2017, Elsevier. d) In situ AFM Reproduced with permission^[^ ^135^ ^]^ Copyright 2018, RSC Publishing e) ETEM‐AFM. Reproduced with permission^[^ ^143^ ^]^ Copyright 2020, American Chemical Society. f) In situ XRT Reproduced with permission^[^ ^144^ ^]^ Copyright 2014, RSC Publishing g) Electrode stress monitoring cell, Reproduced with permission^[^ ^145^ ^]^ Copyright 2021, American Chemical Society. and h) In situ XAS Reproduced with permission^[^ ^146^ ^]^ Copyright 2021, European Chemical Societies Publishing.

Optical microscopy is one of the most common and cost‐effective techniques for analyzing the sodium‐ion deposition behavior and sodium dendrites at a sub‐micron level. It is an older, but still widely used technique for real‐time applications, as shown in **Figure** **20**a. The primary goal of the analysis is to closely monitor the dynamic changes in the anode and its interphases during the cycling process.^[^ ^137^ ^]^ Yamaki and their team proposed a mathematical theory based on optical microscopy and were the first group to observe whisker‐type lithium dendrites. Later, He et al. revisited the topic and demonstrated that the whisker‐type lithium dendrites originate from the substrate during the electrochemical process. They observed that the enormous pressure generated at the root of the SEI layer caused parts of it to gradually disappear. These types of dendrites grew continuously from various points on the surface. In another significant contribution, Rodriguez et al. developed an in situ optical imaging method to monitor sodium ion (Na^+^) deposition. They used a hermetically sealed optical cell coupled with an optical microscope for real‐time observations. Three different types of electrolytes were tested in their study: NaPF_6_ in EC/DEC, NaPF_6_ in PC/FEC, and NaPF_6_ in FEC/DEC. These electrolytes were used in sodium‐ion batteries to investigate their deposition morphologies and gas evolution. Na^+^ was galvanostatically deposited on the surface, and the optical images revealed that loose, porous, and small dendrites were formed in all cases. In case of EC/DEC and PC/FEC electrolytes, the uneven sodium deposits and dendrites dissolved quickly into the electrolyte, resulting in the formation of electrochemically inactive dead sodium species. In contrast, NaPF_6_ in FEC/DEC showed fewer and smaller protrusions, with reduced gas evolution and diminished sodium loss. This improved performance was attributed to the FEC‐based SEI layer, which contained higher amounts of NaF components compared to the SEI layers formed in other solvent systems.^[^ ^67^, ^138^ ^]^

### 5.2 In Situ Scanning Electron Microscopy

In situ SEM is an intermediate observation tool for bridging the resolution gap between optical microscopy and transmission electron microscopy (TEM). It operates with an energy beam range of 500 eV to 30 keV to scan the sample surface and generate high‐resolution images, by detecting the backscattered electrons. In situ SEM requires high‐vacuum conditions to ensure stable electron sources, significantly reducing noise signals caused by background scattering. Also, the vacuum environment prevents interactions between highly sensitive materials, such as lithium or sodium, and atmospheric components such as H_2_O, O_2_, and CO_2_, ensuring accurate observations. Zhang et al. designed a homemade experimental setup that contains electrochemical‐scanning electron microscopy (EC‐SEM) liquid cell Figure 20b to investigate the lithium plating/stripping process in ether‐based electrolytes.^[^ ^139^, ^140^, ^141^ ^]^ The setup consists of two chips; the top chip is made of thin silicon nitride for real‐time observation of dendrite formation, whereas the bottom chip is made of quartz and contains two injection orifices for introducing the electrolyte. A small gap between the two chips is used for observing the plating/stripping process. Both copper and lithium electrodes are patterned and adhered to the top chip, and 60 µL of liquid electrolyte is injected through the orifices. When voltage is applied to the electrodes, lithium grows and dissolves at the electrode edges, which can be observed in real time through the silicon nitride thin window on the top chip. The authors systematically studied the plating/stripping process using three different electrolytes: 1 m LiTFSI in DOL/DME, 1 m LiTFSI in DOL/DME + LiNO_3_ additive, and 1 m LiTFSI in DOL/DME + Li_2_S_8_ additives. The growth of lithium dendrites and the formation of dead lithium were observed in all cases. However, with the introduction of additives such as Li_2_S_8_ and LiNO_3_, the dendrites were significantly smaller and less dense compared to those formed with the pristine electrolyte without additives. This indicates that the introduction of additives improves the stability of the lithium deposition process.

### 5.3 In Situ TEM

TEM provides a significantly higher spatial resolution compared to the optical and atomic force microscopy techniques, making it one of the most powerful tools for material characterization in recent years. TEM is often integrated with auxiliary tools such as energy‐dispersive X‐ray spectroscopy (EDS), electron diffraction (SAED), and electron energy loss spectroscopy (EELS), enabling the atomic‐scale analysis of structural, compositional, and electronic properties. However, it also requires ultrahigh vacuum conditions to protect sensitive samples, emphasizing the need for further advances, so this technology is still in its early stages of development. For instance, Wang et al. used a carbon nanofiber current collector for the real‐time investigation of the nucleation and growth behavior sodium ions during the electrochemical process at nanoscale. Their study employs that in situ TEM and SEM cell setups, as shown in Figure 20c. The results revealed that sodium metal grew and dissolved as nano‐ and microparticles, utilizing the available spaces in the carbon fiber network.^[^ ^142^ ^]^ Notably, Na^+^ was transported more uniformly into the inter‐fiber carbon network without disrupting the SEI layer. This uniform ion transport resulted in a dendrite‐free sodium anode, highlighting the promising potential of this approach. The integration of spectroscopic techniques with in situ TEM provides a comprehensive understanding of both the internal and external structures of materials. This approach provides valuable insights into sodium metal anodes and facilitates the development of next‐generation battery technologies.

### 5.4 In Situ AFM

In situ AFM is another impressive tool for analyzing nanoscale features on the surfaces of electrodes and their SEI layers. It can also visualize chemical reactions occurring within a battery system, as shown in Figure 20d. This technique uses van der Waals forces between the probe and the sample surface to determine the topography and mechanical, electrical, and electrochemical properties of SEI layers. Compared to optical microscopy and TEM, in situ AFM provides superior resolution.^[^ ^135^ ^]^ For instance, Chen and his team designed a custom‐made in situ AFM setup that included a gold‐coated planar electrode, O‐ring, AFM probe, and an optical camera to study sodium‐ion deposition in carbonate‐based electrolytes. They used 1 m NaClO_4_ in EC/PC (1:1 ratio) liquid electrolyte. The study investigated three critical factors: nucleation, growth, and the formation of sodium dendrites. The obtained image showed that the first step involved nucleation, occurring at the start of the sodium‐ion deposition process (after 100 s). After 300 s of deposition, smaller sodium dendrites could be clearly observed. When the deposition process was further extended to 1000 s, numerous sodium dendrites and dead sodium species were observed. In contrast, the use of FEC‐containing electrolytes resulted in a more uniform morphology and a higher‐modulus SEI layer.

### 5.5 Environmental Transmission Electron Microscopy–Atomic Force Microscopy (ETEM–AFM)

As shown in Figure 20e, ETEM–AFM is a powerful and advanced hybrid tool that provides real‐time insights into Na dendrite growth. This combined technique enables researchers to simultaneously study the electrochemically plated Na^+^ deposits, SEI layer formation, and the mechanical properties of sodium dendrites. ETEM–AFM observations indicate that sodium deposits exhibit diverse morphologies, including nanorod, nanocube, and nanospherical structures, all of which are classified as dendrites.^[^ ^143^ ^]^ These structures are covered by an SEI layer thicker than 20 nm, primarily composed of Na_2_CO_3_, which plays a crucial role in stabilizing the growing dendrites. Additionally, the mechanical strength of the dendrites is significantly higher, ranging from 36 to 203 MPa, compared to that of bulk sodium. This hybrid setup has proven to be far more effective and informative than the individual techniques alone, providing a comprehensive understanding of the behavior of sodium dendrites and the properties of the associated SEI layer.

### 5.6 In Situ X‐Ray Tomography (XRT)

XRT is a non‐destructive tool that enables the changes in materials and their interphases and also observe the 2D/3D images at a microstructural level, without causing damage. This technique is particularly useful for visualizing sodium metal deposits. XRT device is shown in Figure 20f. Peter and his team developed a Li/Li symmetric cell with polymer electrolytes to study dendrite formation.^[^ ^144^ ^]^ By introducing powerful synchrotron radiation in an in situ XRT setup, the image resolution was enhanced to ≈15 nm. This high resolution makes it easier to identify metals, electrolytes, and their interphases for both lithium and sodium systems. To perform this analysis, the sample is rotated perpendicularly to the incident beam, while multiple 2D images are collected without disrupting the object. These 2D images can later be reconstructed into 3D models using digital geometry software. This technique holds a great potential for investigating the deposition behavior of sodium metal in future studies.

### 5.7 In Situ Electrode for Stress Monitoring

In general, metal batteries face critical issues related to the phobic properties of Li/Na metal anodes, which cause significant volume expansion and contraction during plating and stripping. During subsequent cycles, the substantial volume changes in the sodium/lithium metal structural changes lead to the breaking of its SEI layer, weakening it over time. When the SEI layer breaks, fresh sodium/lithium metal comes into continuous contact with the electrolyte, resulting in side reactions that shorten the lifespan of the battery. Volume expansion at the anode is a major source of stress, which contributes to several issues and requires focused solutions. While many studies have concentrated on dendrite growth, limited research has been conducted on stress generation. Liang and his team experimentally investigated the stress generation mechanism of lithium metal anodes using a specially designed electrochemical mechanical coupled device, as shown in Figure 20g. Based on their findings, they identified failure mechanisms in lithium metal under stress.^[^ ^145^ ^]^ Their experiments revealed three key insights: the increased stress in lithium metal primarily depends on the amount of lithium deposited (areal capacity), not on how fast lithium ions are transferred (current density). In addition, they investigated different current densities and found that the stress is directly proportional to the volume of lithium deposits. Over time, the lithium anode develops high stress and undergoes permanent expansio, because of repeated changes in its volume during plating and stripping. It creates several porous and dead lithium regions, which no longer participate in battery reactions. Once the battery short‐circuits, the stress stabilizes and becomes constant, indicating the safety of the battery.

### 5.8 X‐Ray Absorption Spectroscopy (XAS)

As shown in Figure 20h, XAS is an advanced tool for studying the structural properties of specific elements in a material by passing X‐rays through a sample during cycling.^[^ ^146^ ^]^ Compared to XRD, XAS provides deeper insights, particularly for in situ analysis, enabling researchers to investigate ion and electron transport, redox reactions, phase changes, and SEI layer formation at the interface in real time. However, XAS has certain limitations and is highly sensitive to the sample preparation and experimental parameters, which can lead to artifacts and misleading results. The major issues include the following: 1) if a sample absorbs an excessive dose of X‐rays at a certain time, their high concentration may result in the distorted signal, making it impossible to provide accurate results, 2) if the sample is not prepared uniformly, its interaction with the X‐rays will also be uneven, in such cases, it becomes difficult to distinguish the real surface from spurious products 3) the sample exposure to X‐rays for an extended period can alter its chemical states, leading to possible oxidation or reduction, which may lead to misleading results. Despite these drawbacks, XAS remains a valuable technique for real‐time analysis, provided that special attention is paid to sample preparation and experimental conditions to minimize artifacts and ensure reliable results.

## 6 Understanding Deposition Behavior Using Cryogenic Tools

**Figure 21:**
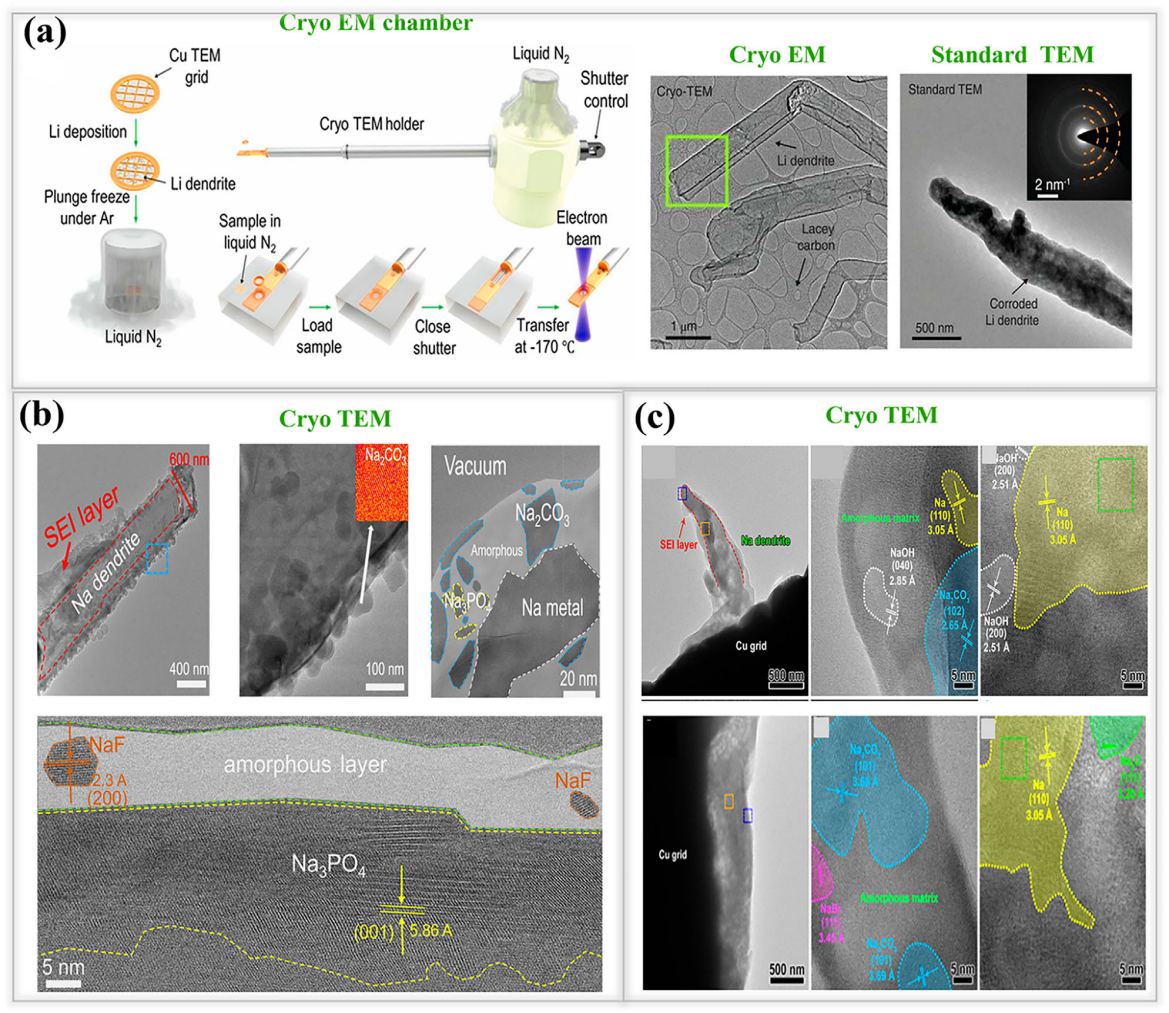
a) Lithium metal dendrites were electrochemically deposited on a Cu TEM grid and preserved in a cryogenic environment. The grid was transferred into a cryo‐EM holder while being maintained under liquid nitrogen; cryo‐EM and standard TEM images. Reproduced with permission^[^ ^177^ ^]^ Copyright 2018, American Chemical Society. b) Dendrites observed by cryo‐TEM analysis using EC:DMC electrolyte without and with FEC additive. Reproduced with permission^[^ ^178^ ^]^ Copyright 2021, Nature Communications Publishing. c) Cryo‐TEM images obtained in NaPF_6_ in diglyme electrolyte without and with CTAB additive. Reproduced with permission^[^ ^179^ ^]^ Copyright 2022, American Chemical Society.

Cryo‐electron microscopy(cryo‐EM) is a powerful analytical technique for studying highly sensitive samples, such as lithium and sodium, which are reactive to air, during transfer to the chamber of typical instruments used in SEM, TEM, or XPS. Also, owing to its low melting point, sodium metal is vulnerable to being damaged by high‐energy electron beams, making it challenging to obtain reliable data for the development of real sodium metal batteries.^[^ ^95^, ^123^, ^147^ ^]^ This approach is particularly effective for providing insights into the formation of the SEI layer and deposition of sodium ions in their original states.^[^ ^148^, ^149^ ^]^ Then, analysis is conducted at cryogenic temperatures, by rapidly freezing the sample using liquid nitrogen or liquid ethane. The rapid freezing process prevents atmospheric reactions and protects the sample from damage caused by high‐energy electron beams, a limitation often encountered with conventional analytical methods. Cryo‐EM enables the investigation of highly sensitive battery materials with enhanced precision, preserving the structure and properties of the material and providing a clearer understanding of the sodium deposition behavior and SEI layer formation.^[^ ^150^, ^151^ ^]^ Initially, Cui and his team developed a Cryo‐EM chamber (**Figure** **21**a) to create a cryogenic environment using liquid nitrogen.^[^ ^177^ ^]^ Lithium ions (Li^+^) were deposited onto a Cu TEM grid under typical battery assembly conditions. Then, the TEM grid was removed from the cell, thoroughly washed with the electrolyte, and rapidly frozen using liquid nitrogen. At the low temperature (−170 °C), lithium metal remained stable, owing to its lack of reactivity with liquid nitrogen or ice. This process preserved the formed dendrites and the related structural information. The experimental results showed that single‐crystal lithium dendrites and their uniformity using Cryo‐EM. In contrast, standard TEM often causes damage to the substrate due to the high dose of electrons.^[^ ^152^ ^]^ After showing the advantages of cryogenic environments, many analytical instruments have been integrated with cryogenic conditions to enhance their capabilities. This development has significantly improved the accuracy and reliability of data on sensitive battery materials, paving the way for further innovation in battery research.^[^ ^153^ ^]^ Meng‐Gu and his team used cryo‐transmission electron microscopy(cryo‐TEM) to investigate the composition and structure of the SEI layer on sodium metal.^[^ ^178^ ^]^ They examined various electrolytes, both with and without fluoroethylene carbonate (FEC) additives.^[^ ^152^, ^154^, ^155^ ^]^ In their experiments, sodium ions were deposited onto a TEM grid placed on Cu foil (Na‐Cu) to study the SEI layer formation. They tested 1 mm NaPF_6_ dissolved in EC:DMC under two conditions: after 1 cycle and after 10 cycles. Without FEC additives, the SEI layer showed significant variations in thickness, ranging from a few to hundreds of nanometers. The SEI was primarily composed of crystalline Na_2_CO_3_, formed through electrolyte reduction, and amorphous Na_3_PO_4_, which appeared nonuniform with varying thicknesses. This kind of SEI had a mosaic‐like structure Figure 21b, but lacked uniformity, leading to uneven ion transport. Over subsequent cycles, the SEI grew unevenly, and in some regions, it broke, exposing fresh sodium metal to the electrolyte. This exposure triggered the formation of new SEI products and, in severe cases, promoted the growth of sharp dendrites capable of reaching the cathode. However, when the FEC additive was added to the electrolyte, dendrites still formed, but were significantly smaller compared to those generated in electrolytes without FEC. Notably, the SEI layer exhibited two distinct regions, a dark crystalline layer (Na_3_PO_4_) in the middle and a light amorphous layer (NaF) on top of the sodium metal. These features were clearly visible through the cryo‐TEM analysis. The uniform SEI layer effectively protected the sodium metal from the electrolyte, and the authors concluded that the FEC additive significantly improved the battery performance. Li and his team also utilized cryo‐TEM to investigate the microstructure of the SEI layer and the effects of additives on the sodium metal. Without additives, the SEI layer formed on sodium was nonuniform, with varying thicknesses and inconsistent SEI components. Also, sodium dendrites were observed to grow individually, reaching heights of ≈500 nm. As shown in Figure 21c, cryo‐TEM images further revealed that the SEI had a mosaic‐like structure, comprising an amorphous NaOH domain in the outer region and a crystalline Na_2_CO_3_ inner region, along with metallic Na and NaF components derived from the electrolyte. Upon the addition of CTAB to the diglyme electrolyte, significant morphological improvements were observed in the deposited Na. The CTAB additive facilitated the formation of a more uniform and stable SEI layer, such as Na_2_CO_3_ and NaBr, dispersed in an amorphous matrix, along with NaOH, Na_2_O, and NaF. This SEI layer was more uniform and stable than that formed without additives, effectively improving the sodium metal surface and suppressing dendrite formation.^[^ ^179^ ^]^

## 7 Technoeconomic Analysis of Sodium Batteries

The increasing demand for consumer electronics, electric vehicles, and large‐scale energy storage devices has spurred the development of new rechargeable secondary batteries. Since their commercialization in the 1990s and the award of the Nobel Prize to Chemistry in 2019, LIBs have become ubiquitous, significantly impacting the energy sector and transforming human lifestyles. However, concerns on the long‐term availability and cost of lithium resources have prompted interest in alternative technologies.^[^ ^81^, ^155^ ^]^ SIBs have emerged as a promising option in this context, owing to the abundance and wide availability of sodium, with global resources estimated at 23 000 ppm, making it ≈400 times more abundant than lithium. This suggests that sodium‐based batteries could provide a more sustainable and cost‐effective solution for meeting growing energy demands.^[^ ^155^ ^]^ In addition to wide material availability, SIBs provide economic advantages in terms of component selection. For instance, aluminum foil can be used as a current collector for both the anode and cathode in SIBs, because it does not alloy with sodium. This contrasts with LIBs, which require more expensive copper foil for the anode. Such material choices contribute to the potential cost reductions associated with the SIB technology. Despite these advantages, SIBs are still in the early stages of development, and comprehensive technoeconomic analyses are necessary to fully assess their viability. So, researchers should focus on evaluating the entire battery system, rather than limiting the analyses to individual components such as electrodes, electrolytes, and separators. This holistic approach will provide a clearer understanding of the potential cost benefits and performance metrics of SIBs compared to LIBs. Several companies have started to develop SIB technologies. For example, Faradion Limited introduced a sodium‐ion battery in 2011, utilizing a hard carbon anode and a high‐voltage cathode in a liquid electrolyte. Their pouch cells achieved an energy density of 140–150 Wh kg^−1^ and exhibited stable performance over 300 cycles at a 3C rate.^[^ ^123^ ^]^ Similarly, Tiamat, founded in France in 2014, employed polyanionic materials to deliver an energy density of 100–120 Wh kg^−1^ at the cell level.^[^ ^155^ ^]^ While these developments are promising, a direct technoeconomic comparison between LIBs and SIBs is challenging, owing to the early‐stage nature of the SIB technology. In conclusion, sodium‐ion batteries represent a compelling alternative to lithium‐ion batteries, primarily due to the abundance and cost‐effectiveness of sodium resources. However, further research and comprehensive technoeconomic analyses are essential to fully understand their potential and address the challenges associated with their development and commercialization.

## 8 Conclusion and Future Perspectives

The ever‐growing demand for electric vehicles, portable electronics, and energy storage devices requires the development of advanced systems with high energy and power densities. As the global population grows, the need for sustainable and affordable energy solutions is also increasing. The limitations of current lithium‐ion battery systems clearly suggest that they may not be sufficient to meet future demands. This challenge has motivated the exploration of alternative systems with cost‐effective and scalable features. Among them, SMBs emerged as a promising alternative owing to the abundance, low cost, and widespread availability of sodium. However, the practical application of sodium metal anodes faces significant challenges, including the instability of the SEI layer, volume expansion/contraction, gas evolution, and dendrite growth. Sodium metal anodes are still in the early stages of development, and addressing these issues is critical to their success. In this review, we have outlined the fundamental problems and working mechanisms associated with sodium anodes, including the dendrite formation mechanism and theory. We have focused on promising surface engineering approaches, with a detailed discussion on artificial (organic, inorganic, and hybrid)SEI layers as well as their formation strategies and electrochemical performances. These artificial SEI layers show great potential in reducing dendrite formation and improving the electrochemical performance of sodium metal batteries. Compared to their natural counterparts, these artificial layers are more stable and effective, as well as relatively easier to prepare. Key factors such as sodium ion diffusion, nucleation, crystal growth, reaction rates, and dendrite growth must be further understood, ideally through direct visual evidence, to ensure the reliability of sodium metal anodes. This review also summarizes various characterization tools, including XPS, XRD, TEM, and FTIR, along with advanced in situ techniques and Cryogenic tools to study the working behavior of sodium anodes in real time. Future research must prioritize the optimization of SEI layer properties, including thickness, homogeneity, and mechanical strength, while simultaneously addressing issues such as volume expansion, gas evolution, and yield strain. For practical applications, it is critical to consider the cost, scalability, reproducibility, and manufacturing conditions, including high‐vacuum environments. Beyond experimental studies, validating theoretical models will be crucial in order to gain a deeper understanding of the fundamental mechanisms governing sodium metal anodes. Along with this, there is a growing consensus that Li/Na metal anodes should be sufficiently thin to meet the practical requirements of high‐energy‐density batteries. Reducing the anode thickness enhances both gravimetric and volumetric energy densities, improves utilization efficiency, and facilitates more reversible metal stripping/plating. However, this thin Li/Na also presents challenges, such as increased interfacial reactivity, SEI instability, and a higher risk of dendrite formation. Therefore, future research should also focus on the rational design of ultra‐thin Na/Li metal anodes, employing strategies like host‐structured current collectors, interfacial engineering, and scalable fabrication techniques to ensure long‐term electrochemical and mechanical stability. In conclusion, this review underscores the importance of collaborative efforts in advancing SMB technology and provides valuable insights for the development of next‐generation energy storage systems (**Tables** **1**, **2**, **3**).

**Table 1:** Overview of various organic artificial SEI layers. (Reproduced with permission).

Artificial SEI	Method	Electrolyte	Current density [mA cm^−2^] and areal capacity [mAh cm^−2^]	Life span [h]	Ref
HCOONa@Na	Reacted with formate acid vapor	1 m NaPF_6_ in diglyme	2, 1	2200	[156]
Na@O_f_‐CNT	Floating catalyst CVD and in situ sodiation	1 m NaSO_3_CF_3_ in diglyme	1, 1	6000	[157]
Poly(DOL)	In situ reaction	1 m NaPF_6_ in TEGDME	1, 1	2800	[90]
Na@Zn‐HCNT	Slurry coating	1 m NaPF_6_ in diglyme	5, 1	1000	[158]
PhS_2_Na_2_	Ex situ coating (p‐DB/S_8_ in THF)	1 m NaPF_6_ in 1:1 EC/PC (v/v)	1,1	800	[159]
Na@carbon paper‐NCNTs	Melting Na on carbon paper	1 m NaPF_6_ in 1:1 EC/PC (v/v)	3, 1	180	[160]
PNF@Cu	Slurry coating	1 m NaPF_6_ in diglyme	1, 2	2100	[117]
TMTD@Na	In situ coating (TMTM‐sulfide)	1 m NaPF_6_ in 1:1 EC/PC (v/v)	0.25, 0.25	1600	[161]
PVDF@Cu	In situ sodiation	1 m NaPF_6_ in diglyme	1, 1	1200	[89]
MOF‐199@Cu	Slurry coating	0.6 m LiTFSI and 0.4 m LiNO_3_ in 2:1 DME/DOL(v:v)	1, 1	300	[82]
Bi(SO_3_CF_3_)_3_@Na	Ex situ coating [Bi(SO_3_CF_3_)_3_ in DME]	1 m NaSO_3_CF_3_ in diglyme	0.5, 1	1000	[162]
Sn@Na‐NPs	Slurry coating and in situ sodiation	1 m NaClO_4_ in 1:1 EC/DEC (v/v) with 5 wt.% FEC	10, 1	600	[163]
PSL(pyroprotein seed layer) @ Cu	spin coating and in situ sodiation	1 m NaPF6 in diglyme	1,0.5	300	[164]

**Table 2:** Overview of inorganic artificial SEI layers. (Reproduced with permission).

Artificial SEI	Method	Electrolyte	Current density [mA cm^−2^] and areal capacity [mAh cm^−2^]	Life span [h]	Ref.
Na/NaSn	Rolling‐pressing Sn powder	1 m NaPF_6_ in DME	2, 2	1600	[165]
Na‐Sn@NaCl	Ex situ coating (SnCl_4_ solution)	1 m NaPF_6_ in diglyme	2, 1	4500	[100]
NaBr@Na	W urtz reaction	1 m NaPF_6_ in 1:1 EC/PC (v/v)	1, 1	250	[95]
Na_3_P@Na	Rolling‐pressing red P powder	1 m NaTFSI in 3:7 FEC/EMC (w/w)	1, 1	780	[101]
Na‐Zn	Ex situ coating (ZnCl_2_ in THF)	1 m NaPF_6_ in diglyme	1, 2	1200	[99]
Na‐Ga	Rolling‐pressing metal Ga	1 m NaClO_4_ in EC/DEC (v/v) with 5 wt.% FEC	1, 1	666	[166]
Na_3_PS_4_	In situ P_4_S_16_ solution coating	1 m NaPF_6_ in 1:1 EC/PC(v/v)	1, 1	270	[167]
Na_3_Sb@NaF	Ex situ coating (SbF_3_‐DMC solution)	1 m NaPF_6_ in diglyme	2, 5	2400	[39]
Na_2_Te	Painting nanosized Te powder	1 m NaClO_4_ in 1:1 EC/DEC (v/v) with 5 wt.% FEC	1, 1	700	[168]
Na‐Sn‐Te	Rolling SnTe powder	1 m NaClO_4_ in 1:1 EC/DEC (v/v) with 5 wt.% FEC	1, 1	1390	[169]
3 wt.% SnF_2_@Na	In situ coating (SnF_2_ in DMC)	1 m NaPF_6_ in 1:1 EC/DMC (v/v)	0.25, 0.125	700	[93]
NaH@Na	Electrolyte modification	1 m NaBH_4_ in diglyme	1, 1	1200	[170]
Na_2_S/V@Na	Rolling‐pressing V_2_S_3_ powder	1 m NaClO_4_ in 1:1 EC/DEC (v/v) with 5 wt.% FEC	0.5, 0.5	1000	[3]
Al_2_O_3_@Na	Low‐temperature PEALD	1 m NaClO_4_ in 1:1 EC/DEC (v/v)	0.5,1	120	[108]
Na_3_OCl/Na_3_Bi@Na	Rolling‐pressing BiOCl‐Cu	1 m NaClO_4_ in 1:1 EC/DEC (v/v) with 5 wt.% FEC	1, 1	700	[114]
Na_2_Se/Na_15_Sn_4_@Na	Rolling‐pressing homemade SnSe powder	1 m NaClO_4_ in 1:1 EC/DEC (v/v) with 5 wt.% FEC	0.5,1	2400	[102]
NaN_x_O_y_/Na_3_N@Na	Rolling‐pressing NaNO_3_	1 m NaClO_4_ in 1:1 EC/DEC (v/v) with 5 wt.% FEC	0.5, 0.5	600	[103]
NaI@Na	In situ coating (iodopropane)	1 m NaClO_4_ in 1:1 EC/DEC (v/v)	0.25, 0.75	500	[38]

**Table 3:** Overview of hybrid artificial SEI layers (Reproduced with permission).

Artificial SEI	Method	Electrolyte	Current density [mA cm^−2^] and areal capacity [mAh cm^−2^]	Life span [h]	Ref.
Cu/graphene/PMMA	CVD on Cu foil	1 m NaPF_6_ in 1:1 EC/DMC (v/v)	2, 3	300	[171]
NaCl/Sn/organic@Na	SnCl_2_ and 4‐chloro‐2,6‐dimethylphenol in THF	1 m NaSO_3_CF_3_ in diglyme	4, 4	2000	[118]
NaF/C_4_F_9_SO_2_Na	Immersion in C_4_F_9_SO_2_	1 m NaPF_6_ in diglyme	2, 1	2500	[172]
Al_2_O_3_/PVDF‐HFP@Na	Rolling‐pressing Al_2_O_3_ polymer slurry	1 m NaClO_4_ in 1:1 EC/PC (v/v)	0.5, 2	508	[173]
Na_3_Ti_5_O_12_‐MXene	Slurry coating and in situ sodiation	1 m NaPF_6_ in diglyme	3, 3	400	[174]
IOHL‐Na	Rolling and pressing	1 m NaClO_4_ in 1:1 EC/DEC(v/v) with 2 wt.% FEC	0.1, 0.1	1600	[121]
Sn/C@Cu	Slurry coating and in situ sodiation	1 m NaSO_3_CF_3_ in diglyme	1, 1	4080	[175]
NaF/C‐F/C═C	Rolling‐pressing PVDF on Na metal	1 m NaClO_4_ in 1:1 EC/DEC (v/v) with 5 wt.% FEC	1, 1	770	[121]

## Conflict of Interest

The authors declare no conflict of interest.
